# Genomic dissection of *Escherichia marmotae* provides insights into diversity and pathogenic potential

**DOI:** 10.1093/ismeco/ycae126

**Published:** 2024-10-25

**Authors:** Ulrike Binsker, Carlus Deneke, Hafiz Muhammad Hamid, Ashish K Gadicherla, André Göhler, Annemarie Käsbohrer, Jens A Hammerl

**Affiliations:** Department Biological Safety, German Federal Institute for Risk Assessment, Diedersdorfer Weg 1, 12277 Berlin, Germany; Department Biological Safety, German Federal Institute for Risk Assessment, Diedersdorfer Weg 1, 12277 Berlin, Germany; Department Biological Safety, German Federal Institute for Risk Assessment, Diedersdorfer Weg 1, 12277 Berlin, Germany; Department Biological Safety, German Federal Institute for Risk Assessment, Diedersdorfer Weg 1, 12277 Berlin, Germany; Center for quantitative Cell Imaging, University of Wisconsin-Madison, 1525 Linden Drive, Madison, 53706 WI, United States; Department Biological Safety, German Federal Institute for Risk Assessment, Diedersdorfer Weg 1, 12277 Berlin, Germany; Department Biological Safety, German Federal Institute for Risk Assessment, Diedersdorfer Weg 1, 12277 Berlin, Germany; Department for Farm Animals and Veterinary Public Health, Institute of Veterinary Public Health, University of Veterinary Medicine Vienna, Veterinärplatz 1, 1210 Vienna, Austria; Department Biological Safety, German Federal Institute for Risk Assessment, Diedersdorfer Weg 1, 12277 Berlin, Germany

**Keywords:** *E. marmotae*, pan-genome, virulence, One Health

## Abstract

Anthropogenic activities enhance the interconnection of human, animal, and environmental habitats and drive the evolution and inter-niche transmission of bacteria. Clear identification of emerging bacteria and pathogen control is therefore a public health priority. In 2015, the novel *Escherichia* species *Escherichia marmotae* was assigned, but due to the lack of appropriate detection and typing technologies, the One Health impact of this species is still being unraveled. *E. marmotae* represents a missing link in the impact of *Escherichia* spp. Here, we report 25 *E. marmotae* identified by next-generation sequencing that were previously phenotypically characterized as *Escherichia coli* during national zoonosis monitoring of food-producing animals. Applying fastANI to 153 738 published *Escherichia* spp. genome assemblies, we identified further 124 *E. marmotae*, originally classified as *E. coli*. Phylogenomics of all 149 isolates reveals an undefined population structure that is independent of the ecological niche. We highlight the phenotypic, genomic, and plasmid diversity of *E. marmotae* and provide evidence for gene flow across the species. The latter is illustrated by the acquisition of antibiotic resistance plasmids and pathogenicity islands, such as the type III secretion system. Thus, our comprehensive genomic overview of an emerging potential opportunistic pathogen underlines the importance of improved detection and characterization.

## Introduction

*Escherichia marmotae*, formerly *Escherichia* cryptic clade V (CV), was considered an environmental bacterium due to its isolation from primarily aquatic sources [1, 2]. It was described as a distinct *Escherichia* species based on phylogenetic 16S ribosomal ribonucleic acid gene sequence analyses following its isolation from *Himalayan marmot* (*Marmota himalayana*) feces in 2015 [3]. Since then, the species has been increasingly detected in other ecological niches, representing both animal and human specimens, and some isolates were found to carry antimicrobial resistance (AMR) genes [4–11].

Current surveillance programs for zoonoses, clinical infections, and AMR include a limited selection of bacteria, often collecting only phenotypic data and using high-throughput techniques with only moderate discriminatory power to identify the bacterium of interest. This approach may miss emerging pathogens and novel species that contribute to the dissemination of AMR genes. AMR is a continuously growing threat to global public health, concerning humans, animals, the food chain and the environment [12]. The problematic situation of AMR and zoonotic disease transmission is addressed by the One Health approach, which recognizes the interconnection between the different areas of life [13]. The complexity of AMR and pathogen transmission is further compounded by multiple anthropogenic factors, including urbanization, trade, and travel [14–16]. A key principle in the monitoring of zoonotic and pathogenic agents is the early detection of newly emerging species, which requires accurate phenotypic and/or genotypic approaches, including curated databases. Reliable information on bacterial agents, including their resistance, are essential to address this threat and to support improvements in national and international monitoring concepts, diagnostic approaches and public health actions.

Recently, a number of *E. marmotae* genome sequences have been made available, which included isolates from environmental sources and human infections [9, 17]. The studies identified four isolates from soil and human infections, respectively, and performed pan-genome and core genome analyses on a limited number of isolates in conjunction with published *E. marmotae* genomes. Using different bioinformatic approaches, the studies describe *E. marmotae* as a distinct *Escherichia* species and report initial findings on AMR and virulence genes. However, there is a critical lack of robust knowledge about this species and its implications for One Health. In 2020, there were first indications of the presence of *E. marmotae* in our *E. coli* collection, due to inconclusive low MALDI score values, which are atypical for *E. coli* identification. The isolates were collected as part of the national monitoring program for commensal and pathogenic *E. coli* in food-producing animals. Through detailed investigation using whole-genome sequencing (WGS), we identified 25 *E. marmotae* derived from wildlife and food products that were initially identified as *E. coli* due to the lack of highly discriminatory detection/typing methods for this under-reported species. In an attempt to understand the genomic features and functions of the *E. marmotae* species, and more importantly, to understand the context in which *E. marmotae* is becoming more prevalent, not only as a commensal but also as a potential opportunistic pathogen, we aimed to analyze a comprehensive genomic dataset reflecting the entire species.

In this study, we characterized 25 *E. marmotae* from food and wild boar in Germany, of which 10 isolates exhibited a yet unidentified non-transferable colistin resistance mechanism. We complemented our 25 *E. marmotae* genomes with 124 additional published genome sequences, which were identified by applying fastANI and *in silico* Clermont typing to 153 738 NCBI *Escherichia* spp. assemblies. This allowed us to gain deeper insights into the genomic constitution of the species. Our findings explain previous assumptions of a multifaceted lifestyle of *E. marmotae*, allowing colonization of hosts gastrointestinal tract and survival in the environment.

## Materials and methods

### Background of German isolates

Twenty-five *E. marmotae* obtained from vegetables, meat, and animal feces were collected within the annual national monitoring programs for zoonoses and pathogenic *E. coli* and obtained from different German federal states (Table 1). The strains *E. marmotae* HT073016^T^ (CGMCC 1.12862, DSMZ 28771) and *E. coli* ATCC 25922 (DSM 1103, NCBI 12210) were used as species references (3).

**Table 1:** Metadata of 149 *E. marmotae* isolates analyzed in this study.

Sample	Metadata			ANI^1^		Typing^4^		
ID	Isolation source	Collection date	Isolation country	*E. marmotae* ^2^	*E. marmotae* ^3^	MLST	O-antigen	H-antigen
21-MO00410–0	Wild boar, feces	2016	Germany: Hesse	99.78	98.96	Unknown	Onovel8	H56
21-MO00411–0	Wild boar, feces	2016	Germany: Saxony	99.43	98.86	133	O10	H56
21-MO00455–0	Wild boar, feces	2020	Germany: Mecklenburg-Western Pomerania	99.43	98.90	5600	O2-O50-Gp7/O2-Gp7	H56
21-MO00456–0	Wild boar, feces	2020	Germany: Mecklenburg-Western Pomerania	99.45	98.92	133	O10	H56
21-MO00467–0	Wild boar, feces	2020	Germany: Schleswig-Holstein	99.49	98.93	133	O10	H56
21-MO00471–0	Wild boar, feces	2020	Germany: Rhineland-Palatinate	99.31	98.91	133	O88	H56
21-MO00473–0	Wild boar, feces	2020	Germany: Rhineland-Palatinate	99.47	98.94	Unknown	Onovel8	H56
21-MO00586–0	Wild boar, feces	2016	Germany: Lower Saxony	99.48	98.93	133	O10	H56
21-MO00588–0	Wild boar, feces	2020	Germany: Rhineland-Palatinate	99.50	99.00	2721	O53	H56
21-MO00589–0	Wild boar, feces	2020	Germany: Lower Saxony	99.44	99.18	4104	O130	H56
21-MO00590–0	Wild boar, feces	2020	Germany: Lower Saxony	99.41	98.94	5600	O2-O50-Gp7/O2-Gp7	H56
21-MO00591–0	Wild boar, feces	2020	Germany: Lower Saxony	99.44	99.10	7630	O156	H56
21-MO00592–0	Wild boar, feces	2020	Germany: Saarland	99.46	99.04	5600	O2-O50-Gp7/O2-Gp7	H56
21-MO00593–0	Wild boar, feces	2020	Germany: Thuringia	99.44	98.99	8370	O2-O50-Gp7/O2-Gp7	H56
21-MO00612–1	Wild boar, feces	2020	Germany: Mecklenburg-Western Pomerania	99.45	98.99	133	O10	H56
21-MO00613–0	Wild boar, feces	2020	Germany: Rhineland-Palatinate	99.42	98.82	133	Onovel13	H56
21-MO00625–0	Wild boar, feces	2020	Germany: Rhineland-Palatinate	99.46	98.91	Unknown	Onovel8	H56
21-MO01153–0	Duck, kidney	2014	Germany: Mecklenburg-Western Pomerania	99.44	99.18	Unknown	Onovel13	H56
21-MO01154–0	Duck, feces	2014	Germany: Mecklenburg-Western Pomerania	99.43	98.94	5260	O103	H56
21-MO01155–0	Wild boar, feces	2016	Germany: Schleswig-Holstein	99.52	99.12	9417	Onovel13	H56
21-MO01156–0	Wild boar, meat	2008	Germany: unknown	99.48	99.14	7630	O156	H56
21-MO01157–0	Leafy green	2011	Germany: Berlin	99.47	98.89	133	O10	H56
21-MO01158–0	Leafy green	2011	Germany: Berlin	99.46	98.89	133	O10	H56
21-MO01160–0	Red deer, meat	2011	Germany: Saxony-Anhalt	99.46	99.14	3747	O98	H56
21-MO01161–0	Red deer, meat	2015	Germany: Saxony-Anhalt	99.38	99.00	5566	O2-O50-Gp7/O2-Gp7	H56
TW09308	Freshwater beach	NA	USA: Michigan	99.30	98.78	6500	Onovel13	H56
KTE11	NA	NA	NA	99.43	99.08	5463	Unknown	H56
KTE52	Human	2010	Denmark	99.48	99.02	3727	Onovel13	H56
KTE96	Human	2009	Denmark	99.37	99.04	5463	Unknown	H56
KTE159	Human	2010	Denmark	99.42	98.88	5260	O103	H56
B116	Human, blood	2012	United Kingdom	99.46	99.05	133	Onovel13	H56
E1118	Freshwater	NA	Australia	99.47	99.03	2721	O53	H56
12b_Esco_HA-NL	Human, rectal swab	2012	Netherlands	99.41	99.11	5463	Unknown	H56
12_Esco_HA-NL	Human, rectal swab	2012	Netherlands	99.46	99.09	5463	Unknown	H56
HT073016	Marmot, feces	2012	China: Qinghai-Tibet plateau	99.04	100	7530	O38	H56
MOD1-EC6158	Duck, feces	2012	USA:AK	99.42	98.91	2721	O53	H56
MOD1-EC6157	Duck, feces	2012	USA:AK	99.42	99.02	Unknown	O2-Gp7	H56
MOD1-EC6162	Duck, feces	2012	USA:AK	99.42	99.00	6499	Unknown	H56

(*Continued*)

**Table 1:** Continued

Sample	Metadata			ANI^1^		Typing^4^		
ID	Isolation source	Collection date	Isolation country	*E. marmotae* ^2^	*E. marmotae* ^3^	MLST	O-antigen	H-antigen
MOD1-EC6154	Duck, choana	2012	USA:AK	99.46	98.82	6528	O103	H56
MOD1-EC6149	Duck, cloacae	2012	USA:AK	99.45	98.99	6499	Unknown	H56
MOD1-EC6144	Duck, cloacae	2012	USA:AK	99.46	99.00	2721	O53	H56
MOD1-EC6099	Pig, pleural cavity	1983	USA:PA	99.35	98.94	Unknown	O103	H56
MOD1-EC6097	Duck, feces	2011	USA:AK	99.36	98.89	5443	Unknown	H56
MOD1-EC5949	Duck, feces	2008	USA:AK	99.46	98.94	2721	O53	H56
MOD1-EC5950	Duck, feces	2008	USA:AK	99.44	98.83	6528	O103	H56
MOD1-EC5948	Duck, feces	2008	USA:AK	99.50	98.84	Unknown	O103	H56
MOD1-EC5462	Goose, feces	1993	USA:NY	99.41	99.09	Unknown	Onovel13	H56
MOD1-EC5449	Water	1993	USA:NY	99.40	99.02	6495	Onovel13	H56
MOD1-EC5438	Water	1991	USA:NY	99.38	99.05	Unknown	Onovel13	H56
MOD1-EC5427	Soil	1993	USA:NY	99.50	99.18	7348	O146	H56
MOD1-EC6163	Duck, feces	2012	USA:AK	99.46	98.89	5443	O10	H56
MOD1-EC6150	Poultry, choana	2012	USA:AK	99.39	98.97	Unknown	O2-O50-Gp7/O2-Gp7	H56
MOD1-EC6147	Duck, cloacae	2012	USA:AK	99.38	98.93	Unknown	O2-O50-Gp7/O2-Gp7	H56
MOD1-EC6153	Duck, choana	2012	USA:AK	99.32	98.86	5443	O10	H56
MOD1-EC6098	Pig, pleural cavity	1983	USA:PA	99.35	98.75	5443	O10	H56
MOD1-EC6100	Pig, pleural cavity	1983	USA:PA	99.45	98.92	2721	O53	H56
MOD1-EC6096	Duck, feces	2011	USA:AK	99.43	98.83	Unknown	O2-O50-Gp7	H56
MOD1-EC5451	Water	1993	USA:NY	99.44	99.01	7989	Unknown	H56
MOD1-EC5426	Soil	1993	USA:NY	99.48	99.12	2559	Unknown	H56
MOD1-EC5110	Duck, cloacae	2006	USA:AL	99.36	98.84	5260	O103	H56
20 412–1	Long-tailed weasel, lymph node	2007	USA:WI	99.38	99.08	8158	O180	H56
SC345	Waterline	2005	USA: St. Louis Clyde watershed of Lake Superior	99.12	98.68	133	O29	H56
SC344	Waterline	2005	USA: St. Louis Clyde watershed of Lake Superior	99.10	98.68	133	O29	H56
SC342	Waterline	2005	USA: St. Louis Clyde watershed of Lake Superior	99.12	98.70	133	O29	H56
SC341	Waterline	2005	USA: St. Louis Clyde watershed of Lake Superior	99.39	99.12	2559	Onovel13	H56
SC337	Waterline	2005	USA: St. Louis Clyde watershed of Lake Superior	99.41	99.11	2559	Onovel13	H56
SC331	Waterline	2005	USA: St. Louis Clyde watershed of Lake Superior	99.14	98.72	133	O29	H56
SC329	Waterline	2005	USA: St. Louis Clyde watershed of Lake Superior	99.43	99.08	2559	Onovel13	H56
SC326	Waterline	2005	USA: St. Louis Clyde watershed of Lake Superior	99.12	98.69	133	O29	H56
SC330	Waterline	2005	USA: St. Louis Clyde watershed of Lake Superior	99.39	99.14	2559	Onovel13	H56
CVM N17EC1081	Poultry, meat (chicken wings)	2017	USA:OR	99.39	99.11	7347	Onovel21	H56
UMB2500_14	Human	NA	USA: Missouri	99.44	99.00	3727	Onovel13	H56
644 129	NA	NA	United Kingdom	99.44	99.09	2559	Onovel13	H56
PSU-0676	Water	2000	USA:NY	99.47	98.92	3727	Onovel13	H56
PSU-0449	Lettuce leaf	2002	USA:OH	99.37	99.04	2559	Unknown	H56
ECOL-18-VL-OH-WA-0026	Wolf	2018	USA:WA	99.33	99.68	9576	Unknown	H56
195 605	NA	2015	United Kingdom	99.38	99.12	5463	Unknown	H56

(*Continued*)

**Table 1:** Continued

Sample	Metadata			ANI^1^		Typing^4^		
ID	Isolation source	Collection date	Isolation country	*E. marmotae* ^2^	*E. marmotae* ^3^	MLST	O-antigen	H-antigen
195 741	NA	2015	United Kingdom	99.44	99.03	5463	Unknown	H56
209 701	Animal	2016	United Kingdom	99.48	99.20	3747	O98	H56
150 966	Human	2015	United Kingdom	99.41	99.03	3747	O98	H56
SN2N-1	Human	NA	USA	99.49	99.11	3727	Onovel13	H56
AMC_597	Human, clinical sample	2014	United Kingdom: Oxford	99.49	99.12	7348	O146	H56
AMC_696	Human, clinical sample	2014	United Kingdom: Oxford	99.42	98.99	5463	Unknown	H56
AMC_764	Human, clinical sample	2014	United Kingdom: Oxford	99.49	99.09	7630	O156	H56
AMC_136	Human, clinical sample	2013	United Kingdom: Oxford	99.43	98.99	5566	O2-O50-Gp7/O2-Gp7	H56
PSU-0866	Duck	2015	USA:AK	99.50	99.10	5600	O2-O50-Gp7/O2-Gp7	H56
PSU-0848	Duck	2015	USA:AK	99.39	98.98	5260	O103	H56
PSU-0845	Avian	2015	USA:AK	99.41	99.11	2559	Unknown	H56
PSU-0852	Duck	2015	USA:AK	99.42	99.02	7500	Unknown	H56
PSU-0839	Duck	2015	USA:AK	99.40	98.94	5600	O2-O50-Gp7/O2-Gp7	H56
E690	Cow, feces	2019	Spain	99.42	99.12	6495	Onovel13	H56
12.2610	Duck	2012	USA:AK	99.41	99.03	6505	O2-O50-Gp7/O2-Gp7	H56
11.1596	Duck	2011	USA:AK	99.39	99.01	6505	O2-O50-Gp7/O2-Gp7	H56
93.0724	Soil	1993	USA:NY	99.47	99.11	2559	Unknown	H56
12.2612	Duck	2012	USA:AK	99.42	98.94	2721	O53	H56
93.1447	Bird	1993	USA:NY	99.41	98.92	5260	O103	H56
14.0993	Duck	2014	USA:AK	99.36	98.89	5443	O10	H56
14.0982	Duck	2014	USA:AK	99.37	98.85	5260	O103	H56
11.1597	Duck	2011	USA:AK	99.41	98.88	5600	O2-O50-Gp7	H56
14.0985	Duck	2014	USA:AK	99.37	98.92	2721	O53	H56
11.1600	Duck	2011	USA:AK	99.44	99.01	6505	O2-O50-Gp7/O2-Gp7	H56
8.2195	Duck	2008	USA:AK	99.45	99.02	2721	O53	H56
BS116-C	Human, rectal swab	2019	Switzerland	99.40	98.99	5463	Unknown	H56
RHB42-C09	Sheep, feces pooled	2017	United Kingdom	99.41	99.16	125	Unknown	H56
RHB24-C12	Sheep, feces pooled	2017	United Kingdom	99.78	98.89	5540	O133	H56
RHBSTW-00814	Freshwater sample from upstream of WWTP	2017	United Kingdom	99.32	98.85	Unknown	O103	H56
RHBSTW-00777	Freshwater sample from upstream of WWTP	2017	United Kingdom	99.48	98.96	Unknown	O103	H56
RHBSTW-00605	Freshwater sample from downstream of WWTP	2017	United Kingdom	99.29	98.96	5566	O2-O50-Gp7/O2-Gp7	H56
RHBSTW-00604	Freshwater sample from downstream of WWTP	2017	United Kingdom	99.31	99.03	5566	O2-O50-Gp7/O2-Gp7	H56
RHBSTW-00265	Freshwater sample from downstream of WWTP	2017	United Kingdom	99.87	98.94	5540	O133	H56
RHBSTW-00263	Freshwater sample from downstream of WWTP	2017	United Kingdom	99.86	98.92	5540	O133	H56
EC245	Wild boar, diaphragm	2017	Italy	99.54	99.15	Unknown	Unknown	H56
EC237	Wild boar, diaphragm	2017	Italy	99.51	99.18	Unknown	O84	H56
EC115	Wild boar, diaphragm	2017	Italy	99.47	99.10	7630	O156	H56
1374a	Bird	2018	Australia: Victoria Koo Wee Rup Yallock Creek	99.51	99.07	Unknown	O139	H56
458 094	NA	2017	United Kingdom	99.41	99.03	5260	O103	H56
311 967	Human	2016	United Kingdom	99.41	99.14	5463	Unknown	H56

(*Continued*)

**Table 1:** Continued

Sample	Metadata			ANI^1^		Typing^4^		
ID	Isolation source	Collection date	Isolation country	*E. marmotae* ^2^	*E. marmotae* ^3^	MLST	O-antigen	H-antigen
Uol_22	Cat, urine	2018	United Kingdom	99.36	98.93	133	O4	H56
Jun 77	Human, urinary tract infection	NA	Portugal: Porto	99.39	98.97	5463	Unknown	H56
C15–3	Poultry, feces	2018	United Kingdom	99.23	99.11	7416	O5	H56
C6–9	Poultry, feces	2018	United Kingdom	99.20	99.05	7416	O5	H56
C9–9	Poultry, feces	2018	United Kingdom	99.13	98.85	5391	Unknown	H56
C21–1	Poultry, feces	2018	United Kingdom	99.28	99.06	7416	O5	H56
C14–7	Poultry, feces	2018	United Kingdom	99.44	99.01	Unknown	Unknown	H56
C8–5	Poultry, feces	2018	United Kingdom	99.38	99.10	Unknown	O128	H56
C5–10	Poultry, feces	2018	United Kingdom	99.18	98.90	Unknown	O159/O5	H56
MVC381	Dog, ear swab	2013	Australia: Melbourne	99.10	98.66	133	O4	H56
MVC186	Dog, expressed milk fluid	2011	Australia: Melbourne	99.49	99.05	7495	Onovel13	H56
MVC382	Dog, ear swab	2013	Australia: Melbourne	99.13	98.74	133	O4	H56
895B	Human, rectal swab	2018	France: Paris	99.53	99.08	Unknown	O103	H56
NCTC8196	NA	1950	United Kingdom	99.35	98.98	5260	O103	H56
NCTC11133	NA	NA	NA	99.50	99.07	3727	Onovel13	H56
H1–003-0086-C-F	Human, blood	NA	France: Créteil	100	99.04	5540	O133	H56
ROAR-43	Marten, feces	2002	France	99.43	98.96	4104	O130	H56
MSB1_5C-sc-2 280 313	NA	NA	NA	99.43	98.96	3727	Onovel13	H56
F1T1–17	Pig, feces	2017	United Kingdom	99.38	99.00	Unknown	O2-O50-Gp7/O2-Gp7	H56
F1T2-S10	Pig, feces	2018	United Kingdom	99.43	99.05	5463	Unknown	H56
F1T2-S9	Pig, feces	2018	United Kingdom	99.42	99.07	5463	Unknown	H56
F1T2-S20	Pig, feces	2018	United Kingdom	99.25	98.97	Unknown	O88	H56
F1T2-S87	Pig, feces	2018	United Kingdom	99.51	99.00	2721	O53	H56
F1T2-S89	Pig, feces	2018	United Kingdom	99.52	99.04	2721	O53	H56
F1T2-S88	Pig, feces	2018	United Kingdom	99.53	99.02	2721	O53	H56
F1T3-S124	Pig, feces	2018	United Kingdom	99.36	98.90	5260	O103	H56
F1T3-S123	Pig, feces	2018	United Kingdom	99.44	99.00	Unknown	Onovel8	H56
HUSEmarmC2	Human, urine	2021	Norway	99.51	99.04	5260	O103	H56
HUSEmarmC3	Human, urine	2021	Norway	99.44	99.06	Unknown	Onovel21	H56
HUSEmarmC1	Human, spondylodiscitis	2021	Norway	99.52	98.89	Unknown	Unknown	H56
HUSEmarmC4a	Human, blood	2021	Norway	99.42	98.97	5500	Onovel21	H56
HUSEmarmC4b	Human, pus	2021	Norway	99.41	98.95	5500	Onovel21	H56

^1^ANI was calculated with the fastANI tool (https://doi.org/10.1038/s41467-018-07641-9, version 1.32).

^2^ *E. marmotae* GCA_902709585.2 as reference.

^3^ *E. marmotae* GCA_002900365.1 as reference.

^4^Typing was performed using the BakCharak pipeline (https://gitlab.com/bfr_bioinformatics/bakcharak, version 2.1.0) and the chewieSnake pipeline (version 3.1.1) with the Enterobase *E. coli* scheme.

NA: not available.

WWTP: waste water treatment plant.

### Bioinformatic identification of *Escherichia marmotae* from public database

In order to identify additional *E. marmotae* in the NCBI GenBank database (https://ftp.ncbi.nlm.nih.gov/genomes/genbank/bacteria/), all *Escherichia* spp. genomes (153 738, date 4 March 2022) assembled as contigs, scaffolds, or complete genomes were downloaded using the tool NCBI-genome-download (https://github.com/kblin/ncbi-genome-download, v0.3.1) followed by *E. marmotae* identification by average nucleotide identity (ANI) calculation, *in silico* ClermonTyper prediction and H-antigen determination, as H56 was proposed as a marker (Fig. S6). The NCBI GenBank assemblies were taxonomically classified at species level by the submitters and according to the taxonomic system at the time of submission. Thus, *E. marmotae* were potentially misclassified as *E. coli* or *Escherichia* spp.

The average ANI of all genomes was calculated with fastANI (https://doi.org/10.1038/s41467-018-07641-9, v1.32). Assemblies that differed in size by >20% from a reference genome were excluded. A total of 153 738 genomes were compared pairwise to 10 different reference genomes representative for the genus *Escherichia* (Table S1).

To determine the phylogroup of all GenBank assemblies, we performed Clermont typing using the ClermonTyper (https://github.com/ABN/ClermonTyping, v3) [18].

In addition, we performed *in silico* serotyping of all assemblies using abricate (https://github.com/tseemann/abricate, v1.0.1) with the provided *E. coli* “EcOH” database [19].

Using the above-mentioned tools, 126 *E. marmotae* GenBank assemblies were identified subsequently subjected to a thorough quality control. Therefore, we assessed GC% and contigs length, including length of N50, N75, L50, and L75, using Quast (https://github.com/ablab/quast/, v2) [20]. Completeness of each GenBank assembly was assessed using Busco (https://busco.ezlab.org/, v5.3.2) [21]. It was required that genomes had 95% of single-copy orthologs. Possible contaminated samples were excluded by requiring no >5% of duplicated orthologs. From 126 *E. marmotae* GenBank assemblies, 125 passed the quality control and one duplicate isolate was removed.

All downstream analyses were performed on a final dataset of 149 *E. marmotae* genomes (25 in-house and 124 database assemblies).

### Genome annotation and pan-genome analysis

Genomes were annotated using bakta (1.4.0, database v3, Table S7) [22]. The resulting GenBank files were input to phispy and roary. The pan-genome analysis was performed using roary (v3.13.0) [23].

### *In silico* characterization of *Escherichia marmotae* isolates

All 149 genomes were thoroughly characterized using the bakcharak pipeline (https://gitlab.com/bfr_bioinformatics/bakcharak, v2.1.0) which runs AMRFinder (3.10.1, database v2021-03-01.1) for detection of AMR genes as well as *Escherichia* point mutations. Plasmids were predicted using Platon (1.4.0). Plasmid incompatibility (Inc) groups, virulence genes, and *fimH* variants were identified with abricate (https://github.com/tseemann/abricate, v1.0.1) against the plasmidfinder database, VFDB, and the *fimH* database (https://bitbucket.org/genomicepidemiology/fimtyper_db, v2022-08-29, using thresholds of minimum coverage of 60%, minimum identity of 95%) [24–27]. Virulence genes were associated to virulence categories as described in the VFDB [28].

An allele based phylogenetic analysis was performed using the chewieSnake pipeline (v3.1.1) with the Enterobase *E. coli* scheme (https://enterobase.warwick.ac.uk/species/index/ecoli) containing 2513 loci [29, 30]. The pipeline runs chewBBACA (v2.0.12) for allele calling and grapetree (v2.2) for the computation of the distance matrix and the minimum spanning tree [31, 32]. The cgMLST typing results showed that at least 90% of the target genes were present in all genomes, with a median of 96% (90%–97%) of the 2513 target genes detected per genome.

All genomes were screened for presence of mobile genetic elements (MGEs; e.g. transposable elements, composite transposons, MITEs) using the tool MobileElementFinder (v1.0.3) [33].

Prophages were annotated using phispy (v4.2.6) and the latest pVOG database [34]. The predicted prophages were aligned to all NCBI GenBank phage sequences. For each putative phage, the best reference with at least 60% query coverage was retained [34, 35].

Genomes that harbored AMR genes were further analyzed using plasmidID (https://github.com/BU-ISCIII/plasmidID, v1.6.4) against the plsdb (v2021_06_23_v2) [36].

Figures were generated using ggplot, R, phandango (v1.3.0), grapetree and iTOL (v6.7.4) [32, 37, 38].

## Results

### Underestimated occurrence of *Escherichia marmotae* in national monitoring samples

As part of programs of the national monitoring for commensal and pathogenic *E. coli*, we collected isolates from the food chain in Germany. Prior to 2021, isolates were routinely identified based on their appearance on McConkey agar (pink colonies), while species confirmation by MALDI-ToF was only performed on a representative subset of isolates (<10%). In 2020, we found that 14 isolates were identified as *E. coli* by standard phenotypic methods, but could not be confirmed as *E. coli* by reliable MALDI-ToF quality scores. A subsequent retrospective analysis of *E. coli* isolates with noticeable poor MALDI scores back to 2008 led to the identification of 11 additional questionable isolates. In-depth analysis using WGS and an updated MALDI-ToF database (updated 2022 by implementation of *E. marmotae* master spectra, Bruker) confirmed the 25 isolates as *E. marmotae*. Isolates were previously recovered from fecal samples of wild boar, meat of wild boar and deer, and vegetables from different geographical locations in Germany, suggesting an interconnection of the wildlife sector and the food chain as a reservoir for the species (Table 1, Table S1).

These preliminary findings, and the inability to positively identify *E. marmotae* with common routine typing techniques, stressed the need to study the species more closely for their characteristics, such as AMR and pathogenic potential, and to compare data from our national isolates with globally available data from public databases.

### Phenotypic and genomic diversity of the national *Escherichia marmotae* population

The German *E. marmotae* isolates were analyzed phenotypically and biochemically against the Chinese *E. marmotae* HT073016^T^ type strain and the *E. coli* ATCC 25922 reference strain. We found considerable heterogeneity among *E. marmotae* isolates, as the German strains differed from the *E. marmotae* HT073016^T^ type strain in four reactions (ß-galactosidase expression, indole production and D-melibiose/L-rhamnose fermentation), a metabolic variability also seen in *E. coli* (95% positive ß-galactosidase expression, 98% positive indole production, and 75% positive D-melibiose fermentation) [39]. This highlights the metabolic diversity within *E. marmotae* and revises the previously postulated biochemical profile as summarized in the Supplemental material section (Table S2, Figs S1–S3) [3]. A comparison between *E. coli* and *E. marmotae* using the phenotypic and biochemic tests confirmed that *E. marmotae* is indistinguishable from *E. coli.*

**Figure 1:**
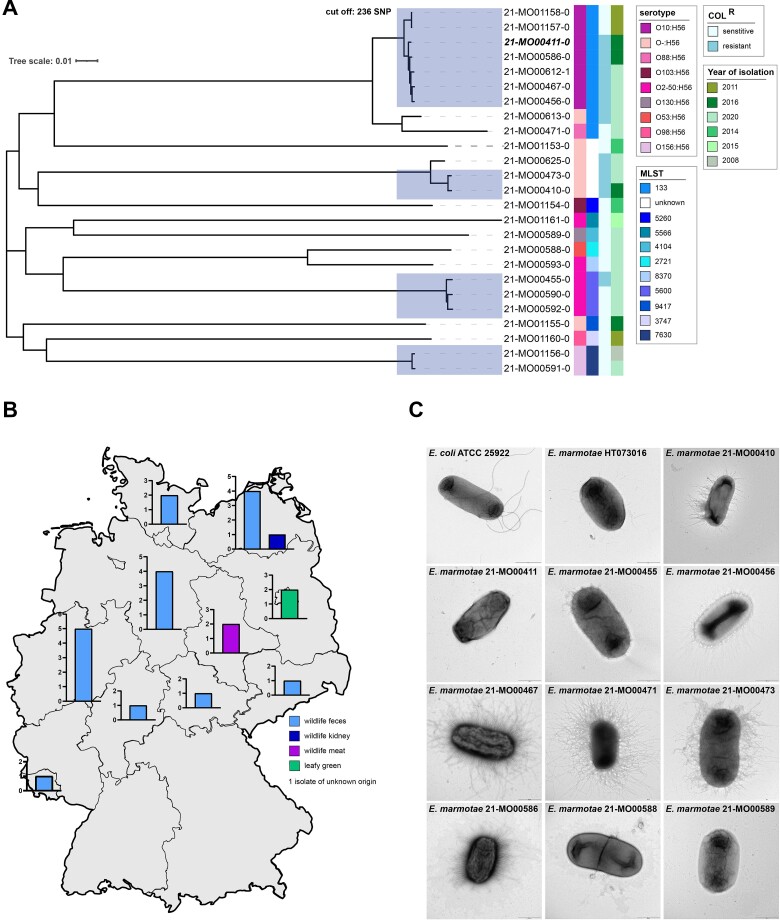
Characteristics of isolates identified as *E. marmotae* collected within national monitoring programs for zoonoses and pathogenic *E. coli.* (A) Maximum-likelihood phylogeny based on whole-genome SNP of 25 isolates using 21-MO00411-0 as reference (bold, italic) and CSI phylogeny 1.4. The isolates covered 77.2% of the reference genome (3 968 676 positions found in the analyzed genomes). Applying a cut-off value of 236 SNP, 4 clusters were identified, highlighted in rectangles. Serotype, MLST, phenotypic colistin resistance of *E. marmotae* and the year of isolation are displayed. (B) Sampling locations by sample type in Germany. (C) Electron microscopy images of selected *E. marmotae* isolates compare to *E. coli* ATCC 25922 (upper left panel) and *E. marmotae* HT073016 (upper middle panel).

Isolates were further characterized by transmission electron microscopy (TEM) and revealed rod-shaped bacteria (Figs 1C, S4). The shape of the German *E. marmotae* were comparable to *E. coli* and to *E. marmotae* reference strains. Interestingly, the German isolates did not seem to express the flagella observed in *E. coli* ATCC 25922 by TEM, but instead presented fimbrial structures.

Phenotypically, 10 of the German *E. marmotae* isolates exhibited resistance to colistin only (Fig. 1A, Table S3) [40]. Neither plasmid-associated *mcr*-genes nor known chromosomal mutations in the *pmrA*/*B* genes were found using *in silico* analysis. In addition, colistin resistance of the *E. marmotae* strains was not transferrable, suggesting for yet unknown mechanism at the chromosomal level.

To understand the extent of the genomic intraspecies diversity across *E. marmotae* from Germany, a single-nucleotide polymorphisms (SNP)-based phylogenetic tree was constructed using the hybrid-assembled reference genome of the local isolate 21-MO00411. The German isolates showed a substantial diversity and covered only 77.2% of the reference genome. The number of SNPs between the strains varied between 68 and 10 541, of which 21-MO00471 showed the largest differences (Fig. S5). Phylogenetic comparison uncovered four distinct clusters of closely related isolates exhibiting identical genoserotypes (serotype) and MLST within clusters (Fig. 1A). The average SNP difference among isolates was 109 in cluster 1 (*n* = 7; range 0–222), 105 in cluster 2 (*n* = 2), 113 in cluster 3 (*n* = 3; range 0–173) and 77 in cluster 4 (*n* = 2). *E. marmotae* within clusters were isolated from different federal states, in different years and from different matrices (Fig. 1, Table 1).

MLST analysis using the typing scheme for *E. coli* reflected the diversity among *E. marmotae* isolates. Ten sequence types (STs) and one unknown ST were determined with ST133 (34.8%) as the most prevalent. For three isolates, MLST could not be determined due to a yet unnotified nucleotide variation in the *adk* gene.

Taken together, the German *E. marmotae* collection showed a greater biochemical and phenotypical similarity to *E. coli* ATCC 25922 than to *E. marmotae* HT073016^T^, which (i) demonstrates the diversity between spatially separated *E. marmotae* (German isolates and Chinese reference strain) and (ii) complicates the differentiation between the two *Escherichia* species in routine diagnostics [41]. The German population also exhibited a high genomic diversity.

### *Escherichia marmotae* pan-genome

ANI analysis and *in silico* Clermont typing of 153 738 published *Escherichia* spp. genome assemblies identified 134 strains that were assigned to *E. marmotae* as they showed ANI values >95.0% and were determined as CV, recently renamed *E. marmotae* (Table S1). Eight strains with ANI values >95.0% with more than one reference, one duplicate isolate, and one isolate of low genome quality (GCA_020555995.1) were excluded from further analysis, resulting in the identification of 124 *E. marmotae* (Fig. S6). In general, ANI values of *E. marmotae* showed greater agreement with a locally close *E. marmotae* H1-003-0086-C-F reference genome (ANI: 99.04%–99.87%, clinical isolate from France) than with a locally distant *E. marmotae* HT073016^T^ reference genome (ANI: 98.66%–99.68%), confirming genetic differences between spatially separated *E. marmotae* (Table 1).

**Figure 2:**
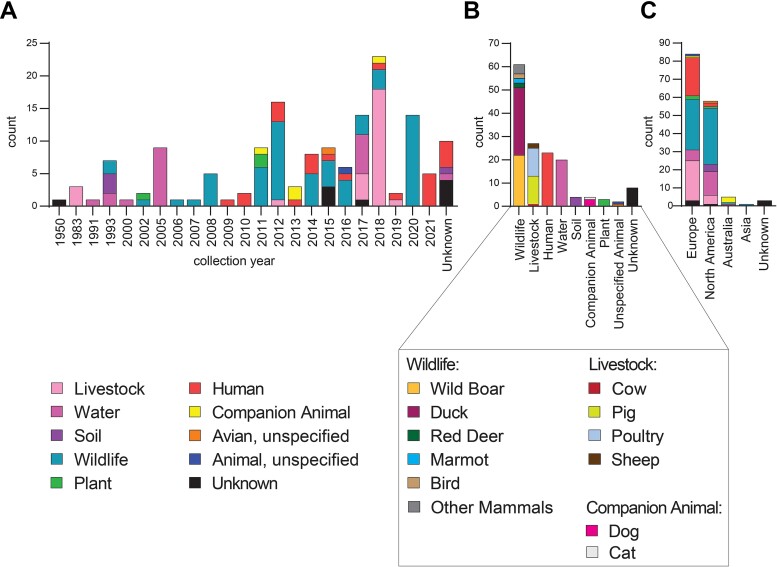
Metadata of *E. marmotae* population. Summary of metadata of 149 *E. marmotae* isolates displayed by collection year (A), sub-epi type according to Supplemental Table S1 (B), and continent (C).

*E. marmotae* were collected from a variety of sources, geographical locations, and over a wide time period (Fig. 2, Table 1). Isolates, including 25 German *E. marmotae*, were collected between 1950 and 2021 from three sources representing animal, environmental, and human isolates sub-divided into 22 niches. The most common niche represented in the collection was wildlife, accounting for 41% of the collection, followed by livestock (18%) and human clinical isolates (15%). Isolates originated predominantly from Europe (58%) and North America (39%).

**Figure 3:**
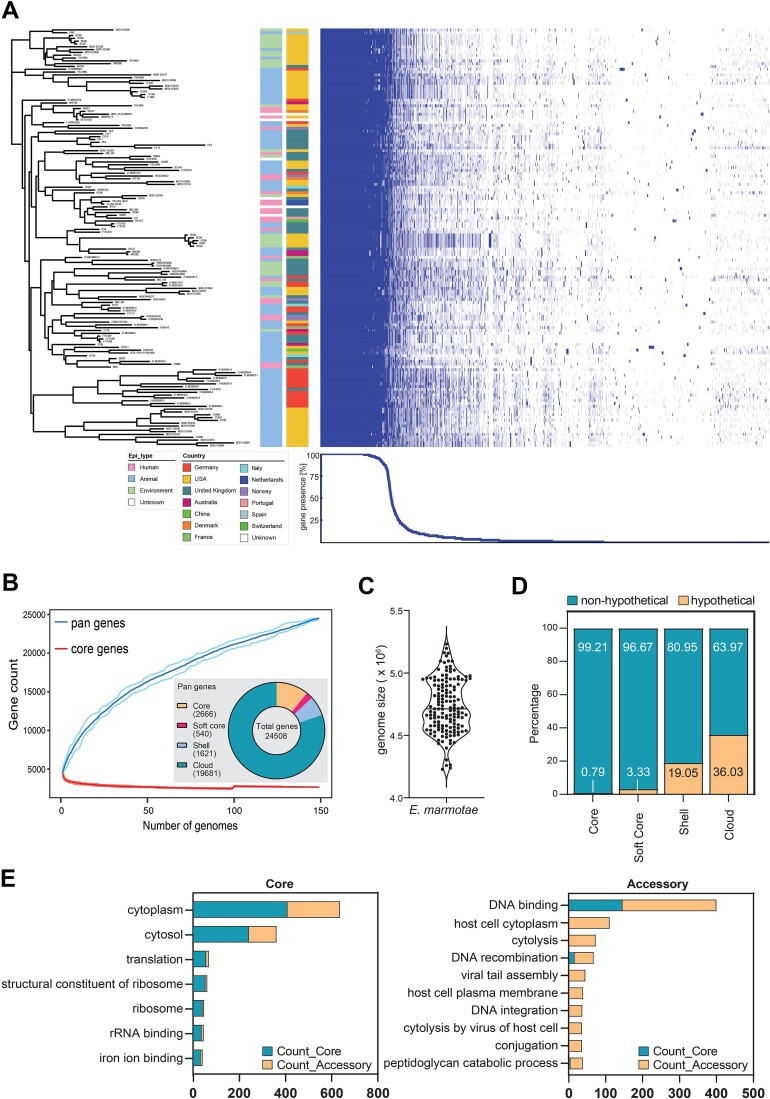
The pan-genome of *E. marmotae*. (A) Data were generated by analyses of whole-genome sequences of 149 isolates. The phylogenetic tree and the presence/absence matrix was constructed using data on 24 508 genes. The gene presence/absence matrix, covering 2666 core genes (present in 148 to 149 genomes) and 540 soft-core genes (present in 141 to 146 genomes), is shown to the right of the phylogenetic tree. Blue, gene presence; white, gene absence. (B) The mean sizes of core and pan-genomes, including minimum and maximum range, relative to the number of genomes added to the gene pool. (C) Variation of genome sizes including the median of 149 *E. marmotae* isolates are shown. (D) Fraction of core, soft core, shell, and cloud genome containing hypothetical proteins. (E) Most frequently occurring GO annotations among the core (core and soft core) and accessory (shell and cloud) genes. Values represent the total sum of genes among all 149 genomes. Statistical significance was determined using a two-sided fisher’s exact test with a multiple-testing correction with a false-discovery rate of 0.05.

To investigate the full genomic diversity of *E. marmotae*, we used the genome assemblies to characterize its pan-genome. We identified a pan-genome of 24 508 gene sequences among the 149 *E. marmotae* genomes (Fig. 3A). *E. marmotae* shared 2666 genes that were present in >99% of genomes, while an additional set of 540 soft core genes was present in >95% of strains. The majority of accessory genes were rare, of which 1621 (6.6%; 15%–95% of genomes) and 19 681 (80.3%; <15% of genomes) genes were included in the shell and cloud genome, respectively. The gene accumulation curve revealed an unsaturated pan-genome by sequencing, indicating an increasing gene pool by adding new *E. marmotae* genomes (Fig. 3B). The average genome size of *E. marmotae* was 4.73 ± 0.21 Mbp (Fig. 3C). Considering the largest contig of each genome assembly, the average GC content of the *E. marmotae* population was 50.03 ± 0.75%.

The mean number of total genes per genome was 4651 genes, of which an average of 1816 ± 266 genes (range, 1254-2520) are part of the accessory genome. In total, 9055 genes occurred only in a single genome, whereas 2380 genes occurred in all genomes. The number of accessory genes was not significantly affected by isolation source (Fig. S7).

The core genome contained a higher proportion of annotated proteins (99.21%) compared with 81% of the shell genes and 64% of the cloud genes (Fig. 3D). The core genome was significantly enriched for seven gene ontology (GO) annotations, including cytoplasm, structural constituent of ribosome and translation, whereas the accessory genome was significantly enriched for GO annotations, such as host cell cytoplasm, viral tail assembly, and conjugation (Fig. 3E).

### The diversity of the *Escherichia marmotae* core genome

**Figure 4:**
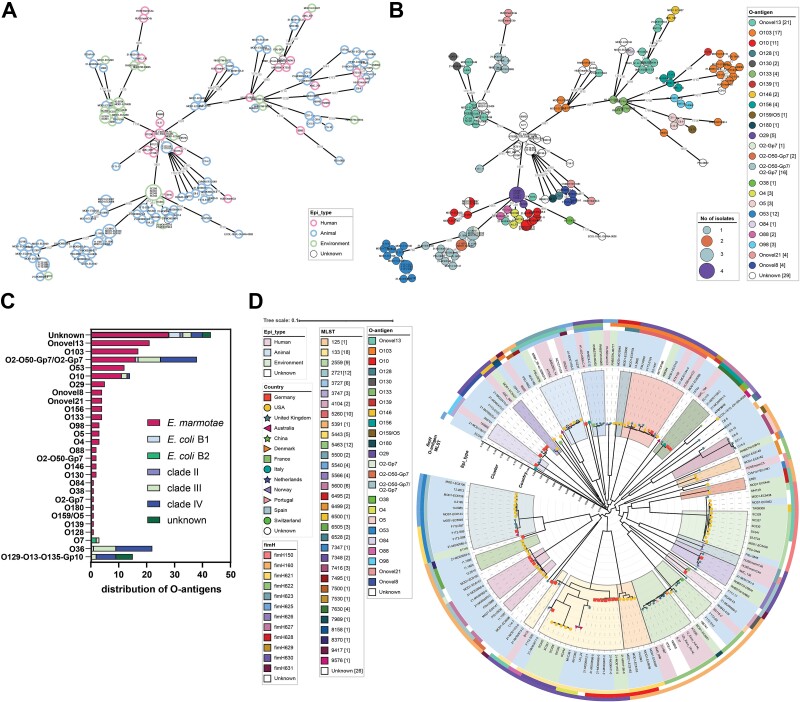
Genomic diversity of *E. marmotae*. (A and B) Minimum spanning tree of 149 *E. marmotae* isolates using the *E. coli* scheme from EnteroBase. Each node represents a distinct cgMLST. The size of each node indicates the number of isolates within that node. (A) Isolates are colored according to their source. (B) Isolates are colored according to their encoded O-antigen gene cluster. (C) O-antigens of isolates carrying the H56 flagellar antigen including non-*E. marmotae* isolates. (D) Phylogenomic relatedness tree based on analysis of SNPs of the genomes of *E. marmotae* using *E. marmotae* HT073016 (GCA_002900365.1) as reference. A total of 62.1% of the reference genome HT073016 was covered by each isolate (3 044 075 positions found in the analyzed genomes). The clusters correspond to the MLST.

We investigated the population structure of 149 *E. marmotae* using a cgMLST scheme for *E. coli* [29]. One hundred and forty-nine isolates were included in the final analysis, resulting in 129 clusters applying a one-allele difference threshold. The majority of clusters contained only one isolate (*n* = 118), while 11 cluster were found containing more than one isolate. A weak clustering of isolates by source was evident, which was largely independent by the country of origin (Figs 4A and S8A). Several clusters of primarily animal, human, or environmental isolates were not only recognized, but also included isolates from other origins (Fig. 4A). Notably, *E. marmotae* are highly clustered with respect to their expressed O-antigen (Fig. 4B). The O-antigen was used as standard for serotyping of *E. coli*. *In silico* serotyping of *E. marmotae* based on somatic and flagellar antigens using *E. coli* as reference organism yielded in 24 different O-antigens with Onovel13 (14%, identity 86.1%–86.3%) being the most frequent followed by O103 (11.3%, identity 87.1%–87.2%), commonly found in STEC causing typically foodborne diseases [42]. Twenty-eight isolates (18.7%) were not-typeable for their O-antigen. Consistent with a previous study, all isolates carried a *fliC*-H56 flagellar antigen (identity 99.69%–100%; Table 1, Table S1) [9]. Notably, we found 61 non-*E. marmotae* isolates encoding the H56 flagellar antigen when analyzing 153 738 *Escherichia* spp. assemblies, demonstrating that H56 is not exclusively present in *E. marmotae* (Fig. 4C). In addition, O/H56-antigen combinations were found that occur in *E. marmotae* and non*-E. marmotae*, such as O2-O50-Gp7/O2-Gp7:H56, whereas other combinations occurred exclusively in non-*E. marmotae*, e.g. O36:H56. However, the H56 antigen in non-*E. marmotae* had a lower nucleotide identity (91.15%–92.53%). Phylogenomics of the H56 protein sequence revealed a distinct clustering that separates H56 from *E. marmotae* from those from non-*E. marmotae* isolates (Fig. S8B). Non-*E. marmotae* isolates had additional non-synonymous mutations distributed throughout the H56 protein sequence compared to *E. marmotae*, making the H56 antigen an insufficient marker and requiring more specific markers for unambiguous identification of *E. marmotae*.

To compare the typing results of cgMLST with other sequence-based methods, an SNP-based phylogenetic analysis was performed. Whole-genome mapping phylogeny based on 10 429 SNPs revealed an overall deep branching between isolates suggesting an evolutionary separation into distinct equally distant lineages (Fig. 4D). In total, 62.1% of the reference genome HT073016^T^ was covered by each isolate. The German isolates did not cluster separately and co-occurred with the international strains. Analysis of the global isolates revealed 22 new MLST types compared to the German population, grouped into a total of 23 MLST clusters. Of these, 14 clusters contained isolates from different sources and 15 clusters comprised isolates from different countries. Comparison with the cgMLST-based minimum spanning tree scheme confirmed a distribution of isolates largely independently of their country of origin or source.

We assessed the genomic diversity of *E. marmotae* within a niche by calculating all pairwise allelic differences (PADs) between their cgMLSTs. With the exception of a few isolates, the majority of *E. marmotae* possessed a large intra-niche diversity with comparable median PADs (Fig. S8C).

The *fimH* allele is another common marker for characterizing *E. coli*. It is used in particular to distinguish the different subclones of the human pathogenic *E. coli* ST131 lineage [43]. Similar to *E. coli*, *E. marmotae* carried a *fimH* allele, which expresses the tip adhesin of type 1 fimbriae mediating mannose-sensitive binding of bacteria to target cells. Twelve different *fimH* alleles and one unknown allele were discovered, of which the *fimH630* allele was predominant with a frequency of 32.2% (Fig. 4D). With the exception of *fimH150*, *fimH160*, and *fimH630*, which were also detected in the *E. marmotae* population studied, the alleles recognized here have not yet been associated with any *Escherichia* species [7, 44, 45]. However, none of the ST131-associated *fimH* alleles were detected in *E. marmotae*. FimH polymorphism is essential for positive selection of different genotypes in different biological niches [46].

### The *Escherichia marmotae* virulome

**Figure 5:**
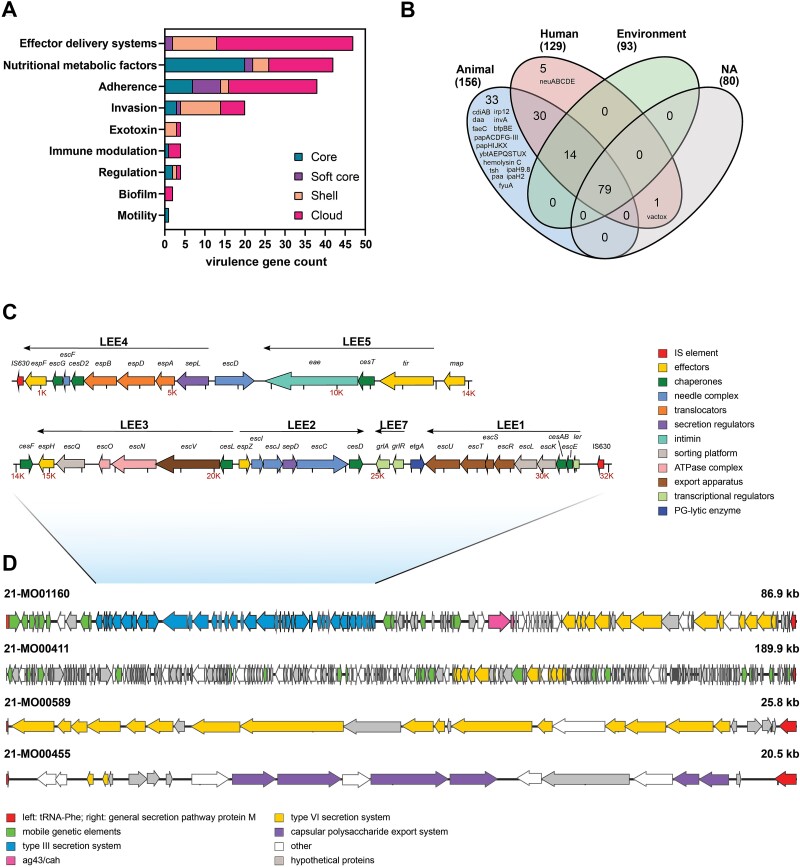
Virulence factors and genomic plasticity of *E. marmotae*. (A) 162 virulence genes were identified in the *E. marmotae* population, comprising nine different virulence factor categories. (B) Distribution of virulence factors in *E. marmotae* isolated from different niches. (C) Genomic organization of the type III secretion system (T3SS, shown here for 21-MO01160) identified in three isolates [21-MO01160, 209 701 (GCA_012546135.1), 150 966 (GCA_012546355.1)]. (D) Genomic plasticity of the T3SS insertion region in select isolates. A region between the phenylalanine tRNA and the general secretion pathway protein M gene was compared between four isolates for which a complete assembled region was available. Hybrid-assembled genomes of 21-MO01160 and 21-MO00411 were used.

Based on the isolation of *E. marmotae* from human specimens and invasive infections, there is mounting evidence that *E. marmotae* appears as both a human pathogen and environmental/commensal bacterium. Content analysis of virulence-associated factors identified 162 different genes in the *E. marmotae* pan-genome, comprising nine distinct virulence factor categories (Fig. 5A, Table S4) Nearly three quarters (71.6%, *n* = 116) of the virulence genes were located in the accessory genome (shell and cloud), of which 15.5% were predicted to be plasmid-derived, such as the invasion protein InvA, the salmochelin siderophore system and components for adhesive fimbriae (Table S4). The majority of virulence-associated genes have been detected in isolates of human and animal origin, of which 5 and 33 were detected only in human and animal strains, respectively (Fig. 5B). Most virulence factors have also been described in various *E. coli* pathotypes (http://www.mgc.ac.cn/cgi-bin/VFs/compvfs.cgi?Genus=Escherichia).

We found a complete type III secretion system (T3SS) in three *E. marmotae*, representing a major virulence factor of enteric pathogens, including EHEC and EPEC (Fig. 5C). The T3SS mediates intimate attachment to intestinal epithelial cells resulting in diarrheal pathology [47]. *E. marmotae* carrying the T3SS were closely related and have been isolated from humans, animals, and red deer meat from Germany and the United Kingdom (serogroup O98; Fig. 3B and D). The T3SSs exhibited high homology between isolates and differed only in the presence of the needle length regulator EscP and a hypothetical protein, thus comprising 39–41 open reading frames. The low GC content of 38.5% compared to the average GC content of *E. marmotae* genomes indicated an uptake of the pathogenicity island (PAI). The T3SS was integrated between a phenylalanine transfer RNA (tRNA-Phe-GAA) and the general secretion pathway protein M gene. The region covered 86.369 bp and included among other multiple MGEs, Ag43/Cah family autotransporter adhesin (WP_024215691.1) involved in biofilm formation, hemolysin expression modulator Hha (WP_000453333.1), a Yew/U toxin-antitoxin system controlling cell division and an entire type VI secretion system (T6SS; Fig. 5D) [48–51]. The region contained in other strains a complete or incomplete T6SS, or genes encoding for capsular polysaccharide export system. The existence of a T3SS and a T6SS PAI in some isolates could indicate a distinct pathotype of *E. marmotae*.

The presence of certain virulence traits in the accessory genome demonstrates the variability of the *E. marmotae* virulome and could be due to the acquisition or loss of accessory genes by either recombination or transmissible elements and horizontal gene transfer (HGT).

### The *Escherichia marmotae* mobilome

**Figure 6:**
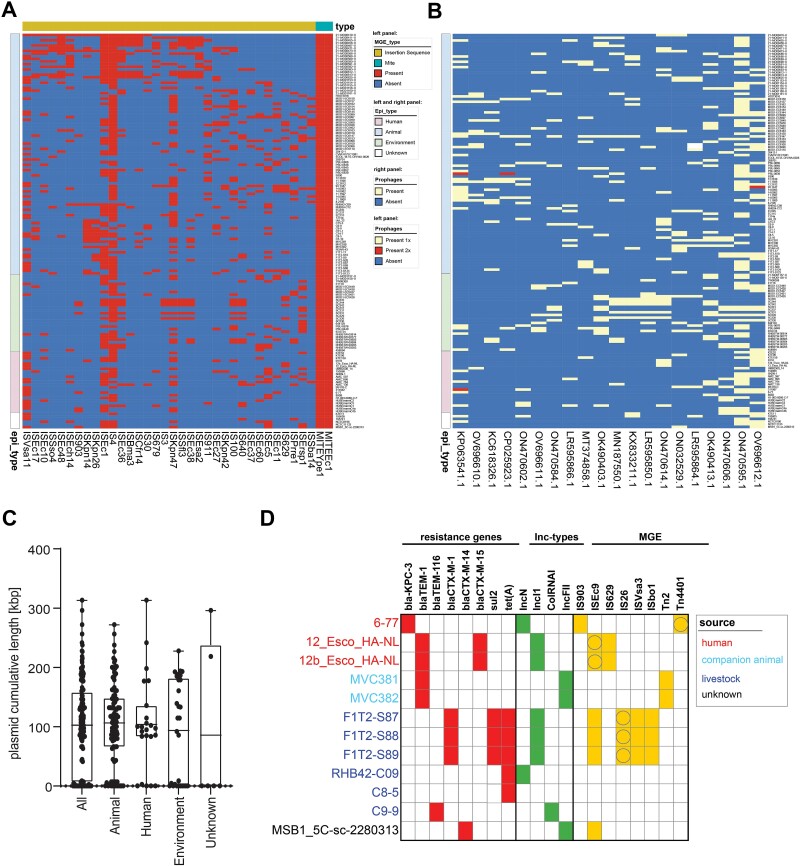
The *E. marmotae* mobilome. (A) MGE were identified using MobileElementFinder (version 1.0.3). Only MGEs are shown which occurred in >10 isolates. (B) Phages were determined by using Phispy and the pVOGs database followed by alignment to all NCBI phage sequences with at least 60% query coverage. Only phages that occur in >10 isolates are shown and “present 1x/2x” means that the prophage is present only once or twice in the same isolate. (C) Plasmid cumulative length in *E. marmotae* from different niches is shown in scatter dot plot and box plot with whiskers from minimum to maximum. Differences in median are not significant as determined by Kruskal–Wallis test followed by Dunn’s multiple comparisons test. (D) Identification of 12 *E. marmotae* carrying antibiotic resistance genes and the corresponding plasmid incompatibility groups (green) using Bakcharak (version 2.1.0). MGEs present in the contig were identified using MobileElementFinder. The MGE associated with the resistance gene contain an additional circle.

Little is known about the HGT and associated MGE and prophages and their contribution to adaptation in different environmental niches. The *E. marmotae* mobilome comprised 331 unique MGEs, including transposable elements (TEs; 55.9%), plasmid incompatibility groups (9.1%), and prophages (35.0%). A total of 2512 TEs were further grouped into composite transposons (8.1%), insertion sequences (ISs; 71.8%), mites (19.7%), and unit transposons (0.4%; Table S5). We mapped the presence or absence of MGEs and prophages shared between isolates from the three sources: human, animal, and environment, and grouped the isolates according to their source. The *E. marmotae* population harbored a variety of TEs that occurred at different frequencies (Fig. 6A). Two mites were ubiquitously present in *E. marmotae*. IS elements such as IS4 (13.0%), ISEc1 (9.4%), and ISKpn47 (8.4%) were relatively common, whereas other IS elements such as IS3 (0.9%) and IS903 (0.8%) were less frequent in the population. There was no apparent clustering of isolates according to their source. However, the German isolates seemed to cluster according to their TE pattern, which could be due to the country of origin itself or the isolation from wild boar. For example, ISEsa2 occurred almost exclusively in German isolates and additionally in EC115 (GCA_014331505.1), which is another wild boar isolate from Italy.

Using Phispy, we identified several prophages in the *E. marmotae* population (Fig. 6B). Temperate phages were most commonly derived from *E. coli*, but phages have also been described in *K. pneumoniae* (OK490413.1; OK490403.1), *Y. pestis* (MT374858.1), and *Salmonella* spp. (ON032529.1; KX833211.1). Isolates harbored an average of six phages, with predominantly 15–17 kbp in size (Fig. S9A and B). The mean GC content of the prophages was 49.8%, which is similar to the GC content of *E. marmotae*, but ranged from 38.7%–59.7%, indicating HGT with different bacterial species as the origin (Fig. S9C).

Plasmidome analysis revealed that the total plasmid content, estimated by the cumulative length, is 102.71 kbp with no significant difference between isolates of different niches (Fig. 6C). The *E. marmotae* population contained 30 plasmid Inc groups, with IncFIB(AP1918) detected in 30.8% of isolates. An average of two Inc groups were found per isolate (Fig. S10). Plasmids are the main vehicle for spreading antibiotic resistance. Twelve isolates were identified carrying an antibiotic resistance gene on a plasmid (Fig. 6D). *E. marmotae* carried one to three genes simultaneously that confer resistance to beta-lactams, sulfonamides, or tetracyclines and were derived from humans, companion animals, and livestock. The resistance genes were located on IncN, IncI1, ColRNAI, and IncFII; all Inc-groups found in other *E. marmotae* isolates and not specific to antibiotic resistance gene carrying isolates. In addition, resistance genes could be associated with IS elements and transposons, such as ISEc09, IS26, and Tn4401. Besides Tn4401, the IS elements were found in other isolates. Using PlasmidID, we identified plasmids with exact or high homology to plasmids described in *E. coli* and *Salmonella enterica* (Table S6). For most of the plasmids, genes for conjugative transfer could be identified.

Analysis of the mobilome uncovered that *E. marmotae* is able to take up exogenous deoxyribonucleic acid presumably to modify its genome and to adapt to the prevailing conditions.

## Discussion

*E. marmotae*, referred to as *Escherichia* CV (atypical *E. coli*) since 2009 and renamed in 2015, was long considered an environmental bacterium [1, 3]. The scientific interest in primarily clinical isolates from humans and economically important animals, and the phenotypic similarity to *E. coli*, undoubtedly led to a knowledge gap about the genetic and functional characteristics of the “novel” species and its impact. In this study, we defined the pan-genome, virulome, and mobilome of 149 *E. marmotae*, including a large number of samples submitted to public databases as *E. coli*, which were largely absent from previous analyses [9, 17, 52].

The identification of only 124 *E. marmotae* genomes from public databases seems low considering the number of over 150 000 *Escherichia* spp. assemblies at the time of the study. It is possible that isolates with deviating results from *E. coli* using molecular phylotyping methods were not subjected to additional analysis by WGS and, as a consequence, the results were not published.

Pan-genome analysis revealed that the genome size of *E. marmotae* varied between 4.2 and 5.2 Mbp, making it slightly smaller on average than *E. coli*, but with a larger core genome [53–55]. Compared to a previous study, the pan-genome of the *E. marmotae* collection in our study increased by >2-fold, reflecting the high genomic diversity within the *E. marmotae* species, while the core genome decreased by 15.8% [9]. The progressive collection of *E. marmotae* genomes may lead to a more precise definition of the core genome, further reducing the size of the core genome and narrowing it down to the most essential genes.

Gene acquisition and loss play significant roles in transitions between commensalism and pathogenicity. Through pan-genomic analyses, we discovered notable genetic variation in virulence factors that have largely only been seen in a few strains. Forty-five percent of the virulence factors identified were infrequent and observed in <5% of the isolates. These included orthologous genes related to those found in pathogenic strains of *E. coli*, such as *tsh* and the *pap* operon, well-studied determinants involved in the pathogenicity of urinary tract infection (UPEC), and the operons of the T3SS and T6SS (enteric *E. coli*) [51, 56–58]. There is certainly genetic variation that might impact *E. marmotae* virulence. However, the presence of virulence factors in *E. marmotae* was predominantly independent from the niche.

Genetic variability was also observed for MGEs, prophages, and plasmids further supporting frequent gene gain and loss across the species. In addition, reports of antibiotic-resistant *E. marmotae* have increased recently [10, 59, 60]. Thus, *E. marmotae* engages in genetic exchange with its environment, allowing it to acquire plasmids carrying resistance genes. However, this raises the question of why antibiotic-resistant *E. marmotae* was not discovered earlier, given that *E. coli* is considered an indicator organism for antibiotic resistance. Could this phenomenon be attributed to the misidentification of antibiotic-resistant *E. marmotae* as antibiotic-resistant *E. coli*, the minimal antibiotic selection pressure in the ecological niche preferred by *E. marmotae*, or is there a bottleneck in the acquisition of resistance plasmids within the *E. marmotae* population? The average plasmid cumulative length per isolate of the 12 antibiotic-resistant *E. marmotae* analyzed in this study is identical to the average plasmid cumulative length of the entire *E. marmotae* population, but is considerably lower than that of antibiotic-resistant *E. coli* [61]. This implies, among others, an inherent restriction in the acquisition capacity of resistance-associated plasmids.

The detection of antibiotic resistance, virulence and other ecologically relevant genes is dependent on the curation of available databases used to compare sequence similarities. It is possible that *E. marmotae* harbors novel resistance mechanisms, such as colistin resistance, and virulence that may not be present in other well-studied bacteria.

Phylogenomics identified a remarkable diversity in the *E. marmotae* population and German isolates intermixed with global isolates. In contrast to *E. coli*, *E. marmotae* has not diversified into distinct lineages. The mixing of similar genomes from different ecological sources, whether food, humans, or the environment, suggests that there is not just one particular lineage that is often associated with each individual source, and that isolates can switch between different environments. This implies a generalist, rather than specialist, lifestyle of *E. marmotae* that is largely independent from adaptive selection. However, our dataset is slightly biased towards animal-derived isolates. In addition, due to the small number of genomes available, additional habitats and adapted *E. marmotae* may have been missed. The inclusion of additional isolates from wider sources, particularly from humans, is important to further extend the population framework, which would allow to eventually separate clones on the basis of their genetic composition. Understanding the frequency, mechanisms and drivers of niche switching in this bacterium is critical to accurately predicting the potential impact of *E. marmotae* in human infections and foodborne diseases, especially in light of the increasing interconnectivity of the human, animal, and environmental sectors due to anthropogenic factors. To achieve this, improved detection methods are a prerequisite for unambiguous identification of *E. marmotae*. The surface antigen H56 has been proposed as a marker for identification, as it has been postulated to be present exclusively in *E. marmotae*. However, our analysis showed that H56 is also expressed by other *Escherichia* spp. Despite the higher identity of H56 in *E. marmotae*, no sequence region could be identified in the gene that was specific to *E. marmotae* and therefore suitable for PCR-based identification.

The data presented here provide a new genomic framework to allow a deeper understanding of the *E. marmotae* population diversity and dynamics. This study will provide a critical foundation and practical support for future studies investigating ecological niche adaptation, pathogenicity and lineage diversification in *E. marmotae*. There is a need for improved phenotypic identification methods, more informed genomic tracking and monitoring of the emergence of virulence and AMR in this increasingly important bacterium.

## Supplementary Material

Supplemental_Material_V12_final_ycae126

Table_S1_Metadata_ycae126

Table_S2_phenotypic_characteristics_25isolates_ycae126

Table_S3_antibiogram_ycae126

Table_S4_Virulence_ycae126

Table_S5_MGE_elements_ycae126

Table_S6_reference_plasmid_predictions_ycae126

Table_S7_Bakta_annotation_ycae126

## Data Availability

We uploaded raw reads and genome sequences into the Sequence Read Archive and GenBank, respectively, at the National Center for Biotechnology Information under Bioproject PRJNA730693 (Table S1).
