# Supplementary material for: Genomic dissection of Escherichia marmotae provides insights into diversity and pathogenic potential
Source: ISME Commun. 2024 Oct 25;4(1):ycae126. doi: 10.1093/ismeco/ycae126 (PMC11546641; doi:10.1093/ismeco/ycae126)
Supplement: Supplemental_Material_V12_final_ycae126 [file supplemental_material_v12_final_ycae126.pdf]

Supplemental Material

Genomic dissection of *Escherichia marmotae* provides insights into diversity and pathogenic potential

Ulrike Binsker[1,*], Carlus Deneke[1], Hafiz Muhammad Hamid[1], Ashish K. Gadicherla[1], André Göhler[1], Annemarie Käsbohrer[1,2] and Jens A. Hammerl[1]

[1]Department Biological Safety, German Federal Institute for Risk Assessment, Berlin, Germany

[2]Department for Farm Animals and Veterinary Public Health, Institute of Veterinary Public Health, University of Veterinary Medicine Vienna, Vienna, Austria

[†]Current address: Center for quantitative Cell Imaging, University of Wisconsin-Madison, Madison, WI, USA

*** Corresponding Author**

Dr. Ulrike Binsker

Address: Unit Epidemiology, Zoonoses and Antimicrobial Resistance, Department Biological Safety, German Federal Institute for Risk Assessment, Diedersdorfer Weg 1, 12277 Berlin, Germany

E-mail: Ulrike.Binsker@bfr.bund.de

Telephone: +49 30 18412 24340

Supplemental Methods

E. marmotae species confirmation and bioinformatics analyses of German isolates

One putative colony of bacterial isolates cultivated on Columbia agar supplemented with 5% sheep blood (Oxoid, Thermo Fischer, Germany) was chosen for species confirmation by MALDI-TOF MS. α-Cyano-4-hydroxycinnamic acid (HCCA, Bruker Daltonics, Bremen) was used as matrix and analyses were performed by MALDI Microflex Biotyper (Bruker Daltonics, Bremen, Germany) as recommended by the manufacturer. MALDI-TOF MS identifications were classified using score values proposed by the manufacturer: a score value between 2.3 and 3.0 indicated very probable species identification; a score value between 2.0 and 2.299 indicated probable species identification. The Compass/BDAL library V11 (no *E. marmotae* identification before 2022) and V12 and higher (*E. marmotae* identification since 2022) were used for species identification.

Short-read WGS was performed using a MiSeq Benchtop sequencer (Illumina, Inc., San Diego, CA, USA) and the MiSeq reagent kit v3 (600 cycles) followed by paired-end sequencing in 2 x 251-bp as previously published (1). Illumina raw reads were trimmed and de novo assembled using the Aquamis pipeline (https://gitlab.com/bfr_bioinformatics/AQUAMIS/, access date: March 2021, May 2021) (2). All samples met the quality criteria as implemented in AQUAMIS, i.e. the samples had sufficient base quality and coverage depth. Genome length and GC content of the assemblies were within the expected range and there was no evidence of contamination. Long-read sequencing was performed with three *E. marmotae* isolates (21-MO00410 and 21-MO00411, 21-MO01160). Therefore, Oxford Nanopore Technology (ONT) sequencing libraries were sequenced on an MinIon Mk1C device followed by data assembly using Unicycler v0.4.8 as previously published (3).

Affiliation of German isolates to *Escherichia* clade V was determined using an *in silico* prediction method according to Clermont (4). Contigs below 200 bp were removed and not included in the calculation.

A phylogenetic tree based on genome-wide sequence variations (single nucleotide polymorphism, SNP) was constructed by using the web-based application CSI Phylogeny 1.4 (https://cge.cbs.dtu.dk/services/CSIPhylogeny/) under default settings (5). To identify SNPs, all input sequences were mapped to the genome of *E. marmotae* 21-MO00411 as reference and screened for relevant nucleotide variations as previously described (6). Based on concatenated alignments of high-quality SNPs, maximum likelihood trees were created using FastTree.

Phylogroup assignment of German isolates by PCR

For phylogroup assignment, a previously described multiplex PCR was conducted with minor modifications (7). The total reaction mixture of 25 µL contained 10 pM of each primer, 12.5 µL of DreamTaq Green PCR Mastermix (Thermo Fisher Scientific, Schwerte, Germany), 6.5 µL of PCR water and 1 µL of the template DNA. An initial denaturation step of 5 min at 94 °C was followed by 33 PCR cycles with 30 s of denaturation at 94 °C, primer binding for 30 s at 57 °C, and 1 min of elongation at 72 °C, as well as a final elongation step of 5 min at 72 °C.

Pulsed-field gelelectrophoresis (PFGE)

Plasmid profiles of the *E. marmotae* isolates were obtained by PFGE using the S1 restriction endonuclease as previously described (8). PFGE was performed using *S.* Braenderup as marker and analysis was conducted with Bionumerics 7.6.3 (Applied Maths, Sint-Martens-Latem; Belgium).

Filter mating and transformation assays

In order to analyze the horizontal gene transfer (conjugation) of colistin resistance, filter mating assays were conducted using the sodium azide (NaN_3)-resistant *E. coli* recipient J53 and colistin-resistant *E. marmotae* at a donor/recipient ratio of 1:2 as previously described (1). In addition, plasmid DNA from colistin-resistant *E. marmotae* was extracted from over-night cultures using the CosMCPrep Plasmid Purification Kit (Beckman Coulter) and transformed

into electro-competent *E. coli* J53. Transformants were selected on LB agar supplemented with 100 mg/L NaN_3 and 1 mg/L colistin sulfate.

Phenotypic characterization

Culture media and cultivation conditions

To assess phenotypic differences of German isolates in comparison to *E. marmotae* HT073016^T and *E. coli* ATCC 25922, all isolates were grown on different selective (Endo and Gassner agar for the verification of *Enterobacterales*; TBX medium to detect ß-D-glucuronidase expression) and non-selective culture media [lysogeny broth (LB) agar, tryptic soy agar (TSA), and Columbia agar supplemented with 5% sheep blood (blood agar)] for 24 h at 37°C.

Analytical Profile Index (API)

The German *E. marmotae* isolates were analyzed with the API® 20E (bioMerieux, Marcy-l'Etoile, France) according to manufacturer's instructions using *E. coli* ATCC 25922 and *E. marmotae* HT073016^T as reference. Profiles were obtained after 24 h and 48 h of incubation at 37°C. ID codes generated from API 20E were used with the apiweb™ and Interpretation Guide (codebook), respectively.

Transmission Electron Microscopy (TEM)

Bacterial suspensions were added to carbon/formvar coated 400 mesh copper grids (3.5 mm diameter; Plano GmbH, Germany), fixed with 2.5% glutaraldehyde and stained with 1% uranyl acetate solution. Imaging was performed on 1400 Plus transmission electron microscope (Jeol GmbH, Germany) operated at 120 kV using a Veleta G2 camera (Olympus, Germany) (9). At least five different areas were observed and used for imaging per grid, with at least four adjacent grid squares per area of observation.

Antimicrobial susceptibility testing (AST)

Susceptibility testing to antimicrobial agents was conducted three times by broth microdilution according to the guidelines of the Clinical and Laboratory Standards Institute (CLSI) using the Sensititre system (EUVSEC3, Trek Diagnostic Systems, East Grinstead, United Kingdom) with a harmonized European panel of antimicrobials (decision 2020/1729/EU) (10).

Supplemental Figures

Figure S1

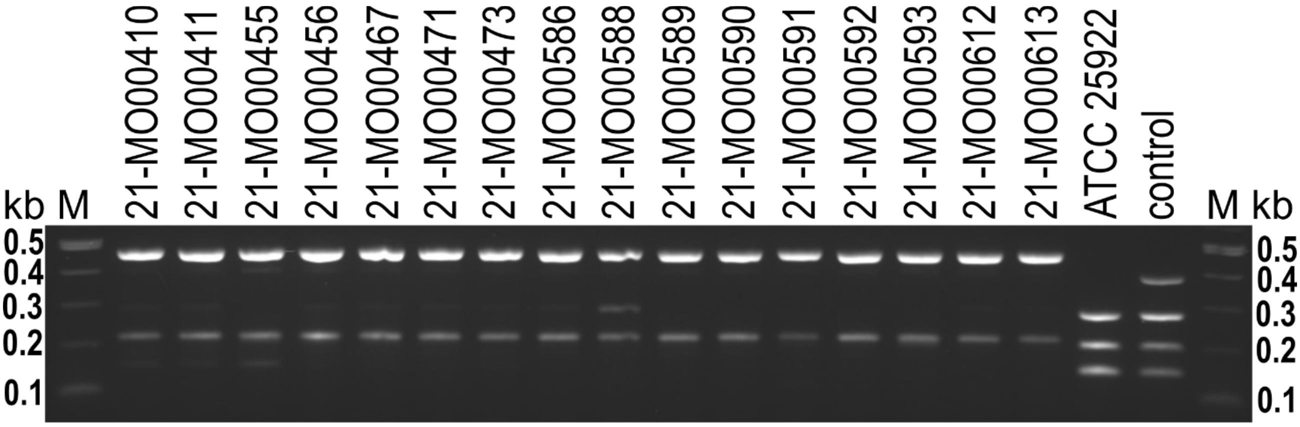

Figure S1: Phylotyping of *E. marmotae* using the *E. coli* phylo-typing method according to Clermont (7). Quadruplex PCR with specific primers for the amplification of the genes TspE4.C2 (152 bp), *yjaA* (211 bp), *chuA* (288 bp), and *arpA* (400 bp). *E. coli* ATCC 25922 served as reference, which is member of the phylogroup B2. A control containing genomic DNA from two different *E. coli* of phylogroup B2 and D was used to represent the four gene bands.

Supplemental Material

Figure S2

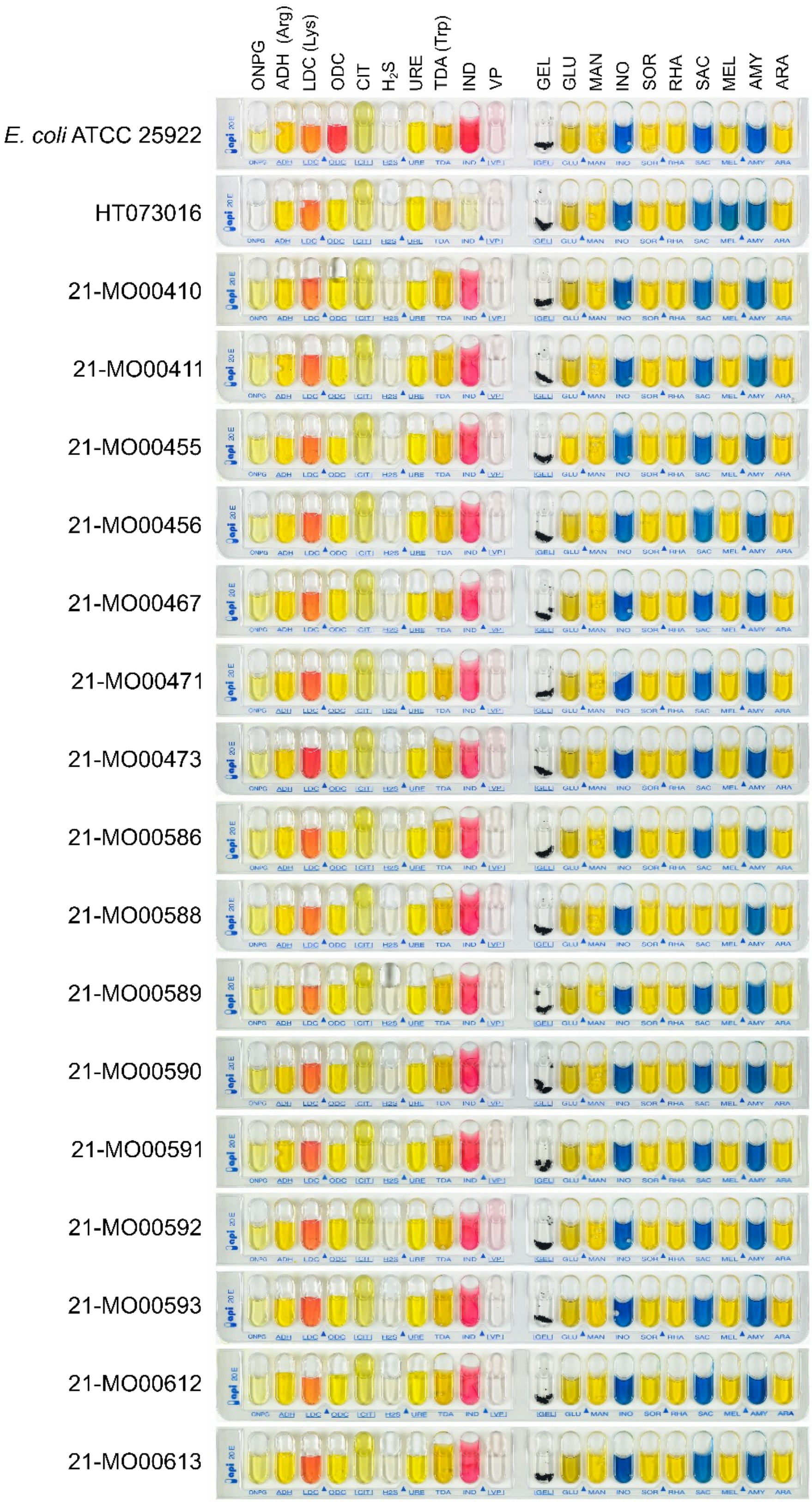

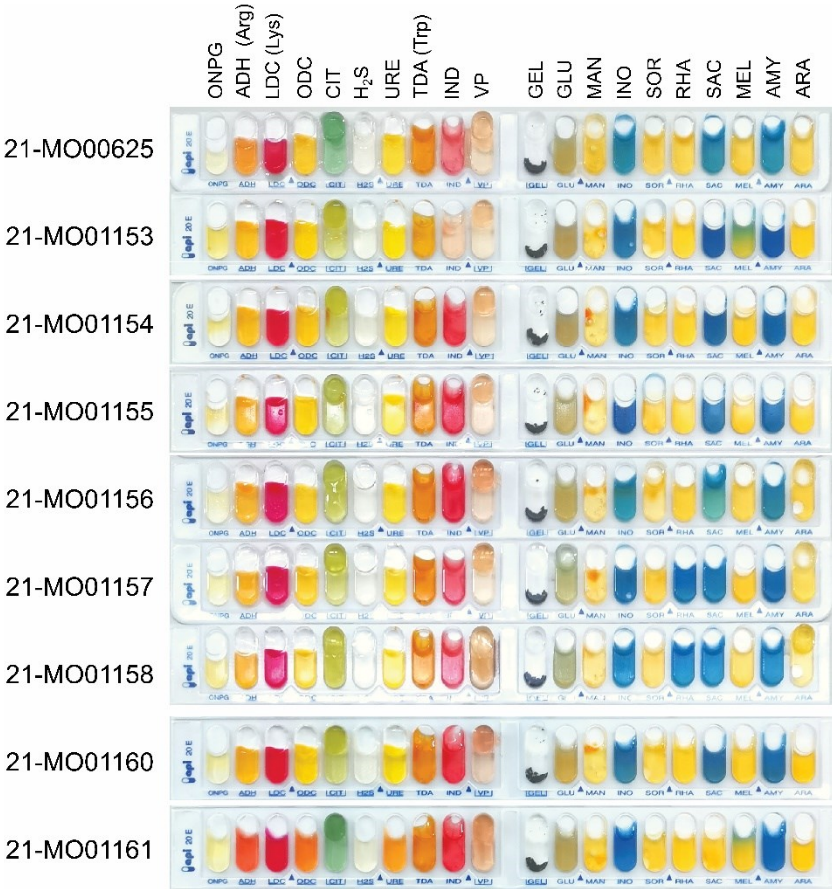

Figure S2: Differentiation of German *E. marmotae* isolates using biochemical reactions. Identification and differentiation of German *E. marmotae* in comparison to *E. coli* ATCC 25922 and *E. marmotae* HT073016 as reference using API 20E strips according to manufacturer's instructions.

Figure S3

A

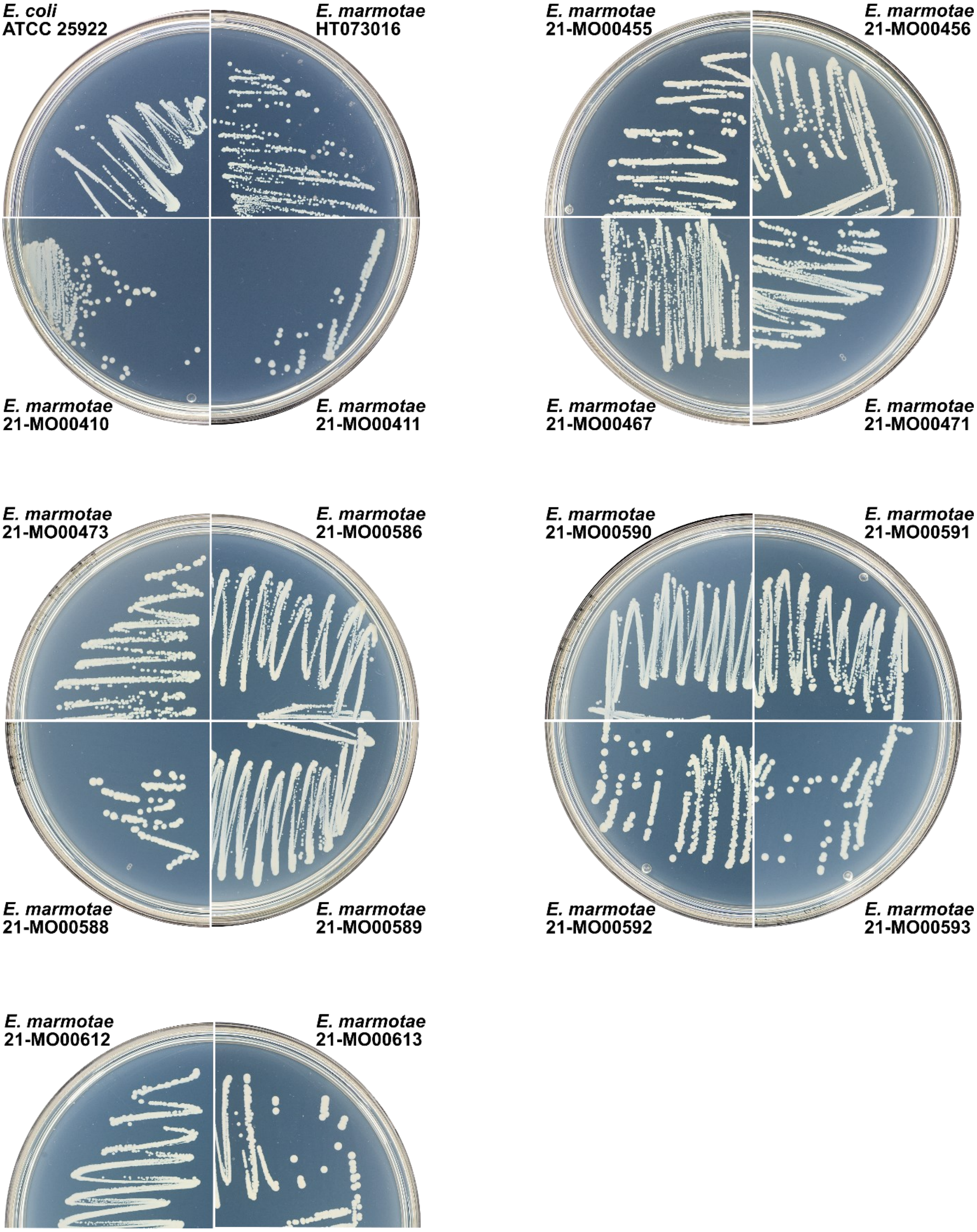
E. coli
ATCC 25922
E. marmotae
HT073016
E. marmotae
21-MO00410
E. marmotae
21-MO00411
E. marmotae
21-MO00455
E. marmotae
21-MO00456
E. marmotae
21-MO00467
E. marmotae
21-MO00471
E. marmotae
21-MO00473
E. marmotae
21-MO00586
E. marmotae
21-MO00588
E. marmotae
21-MO00589
E. marmotae
21-MO00590
E. marmotae
21-MO00591
E. marmotae
21-MO00592
E. marmotae
21-MO00593
E. marmotae
21-MO00612
E. marmotae
21-MO00613

B

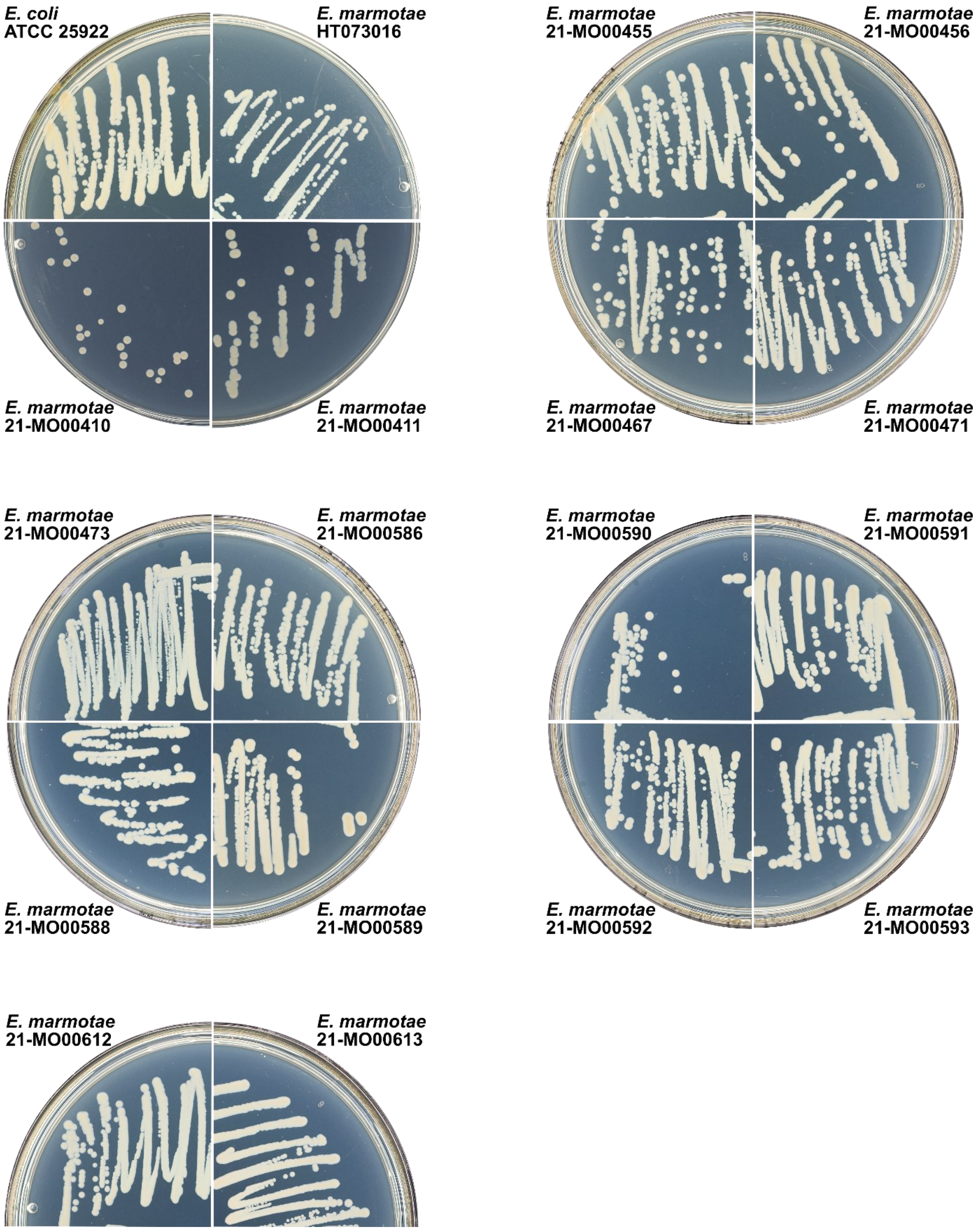
E. coli
ATCC 25922
E. marmotae
HT073016
E. marmotae
21-MO00410
E. marmotae
21-MO00411
E. marmotae
21-MO00455
E. marmotae
21-MO00456
E. marmotae
21-MO00467
E. marmotae
21-MO00471
E. marmotae
21-MO00473
E. marmotae
21-MO00586
E. marmotae
21-MO00588
E. marmotae
21-MO00589
E. marmotae
21-MO00590
E. marmotae
21-MO00591
E. marmotae
21-MO00592
E. marmotae
21-MO00593
E. marmotae
21-MO00612
E. marmotae
21-MO00613

C

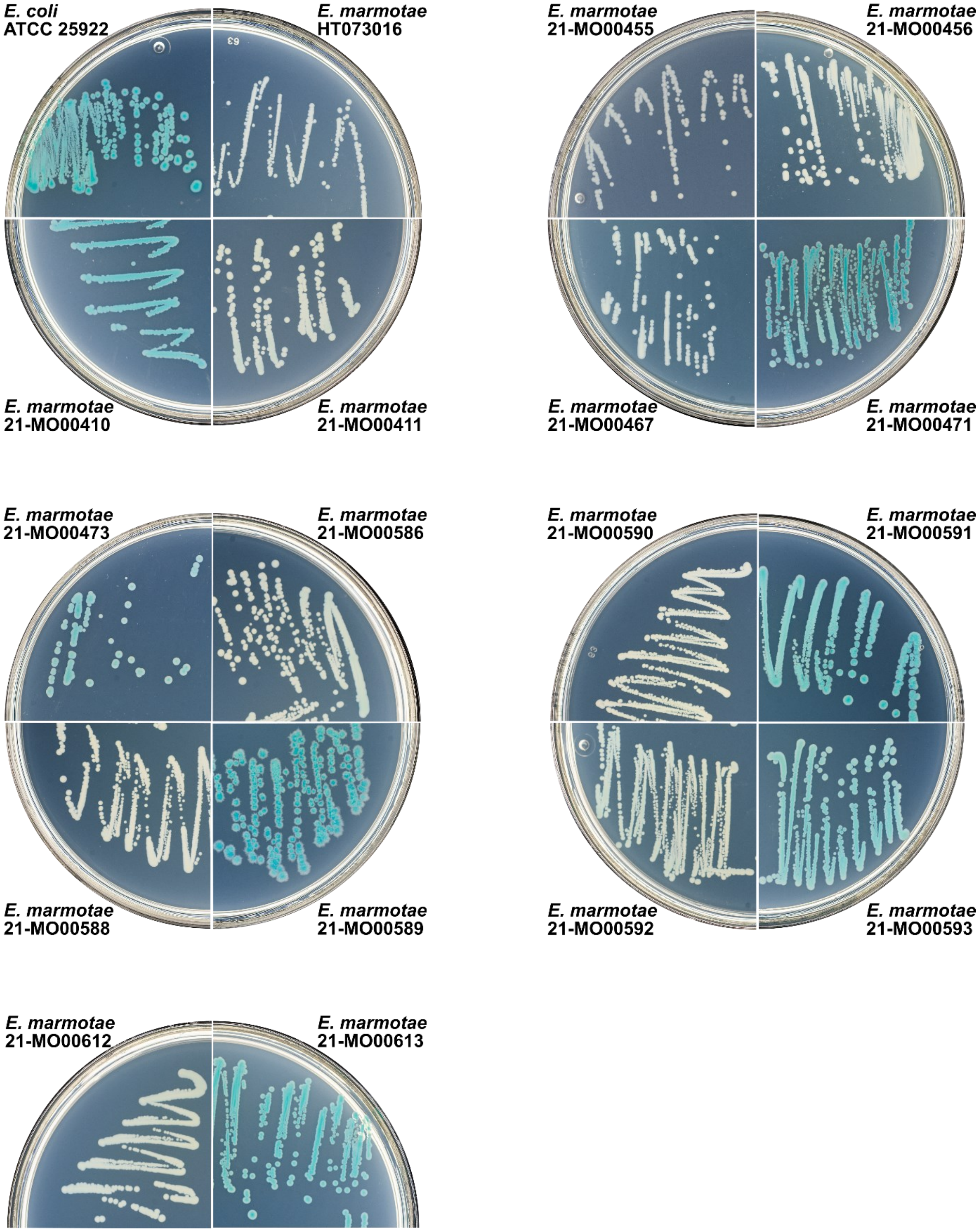
E. coli
ATCC 25922
E. marmotae
HT073016
E. marmotae
21-MO00410
E. marmotae
21-MO00411
E. marmotae
21-MO00455
E. marmotae
21-MO00456
E. marmotae
21-MO00467
E. marmotae
21-MO00471
E. marmotae
21-MO00473
E. marmotae
21-MO00586
E. marmotae
21-MO00588
E. marmotae
21-MO00589
E. marmotae
21-MO00590
E. marmotae
21-MO00591
E. marmotae
21-MO00592
E. marmotae
21-MO00593
E. marmotae
21-MO00612
E. marmotae
21-MO00613

D

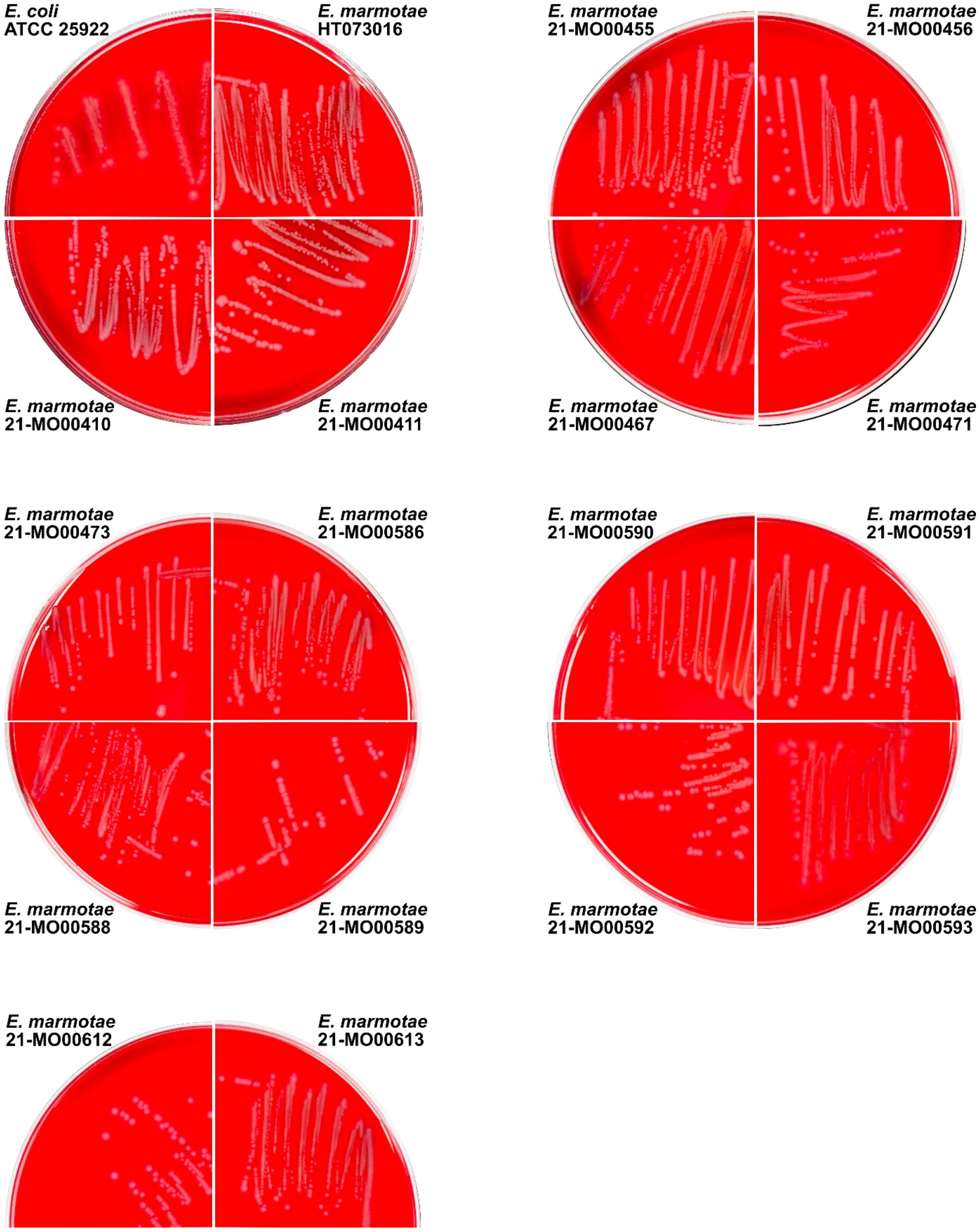
E. coli
ATCC 25922
E. marmotae
HT073016
E. marmotae
21-MO00410
E. marmotae
21-MO00411
E. marmotae
21-MO00455
E. marmotae
21-MO00456
E. marmotae
21-MO00467
E. marmotae
21-MO00471
E. marmotae
21-MO00473
E. marmotae
21-MO00586
E. marmotae
21-MO00588
E. marmotae
21-MO00589
E. marmotae
21-MO00590
E. marmotae
21-MO00591
E. marmotae
21-MO00592
E. marmotae
21-MO00593
E. marmotae
21-MO00612
E. marmotae
21-MO00613

E

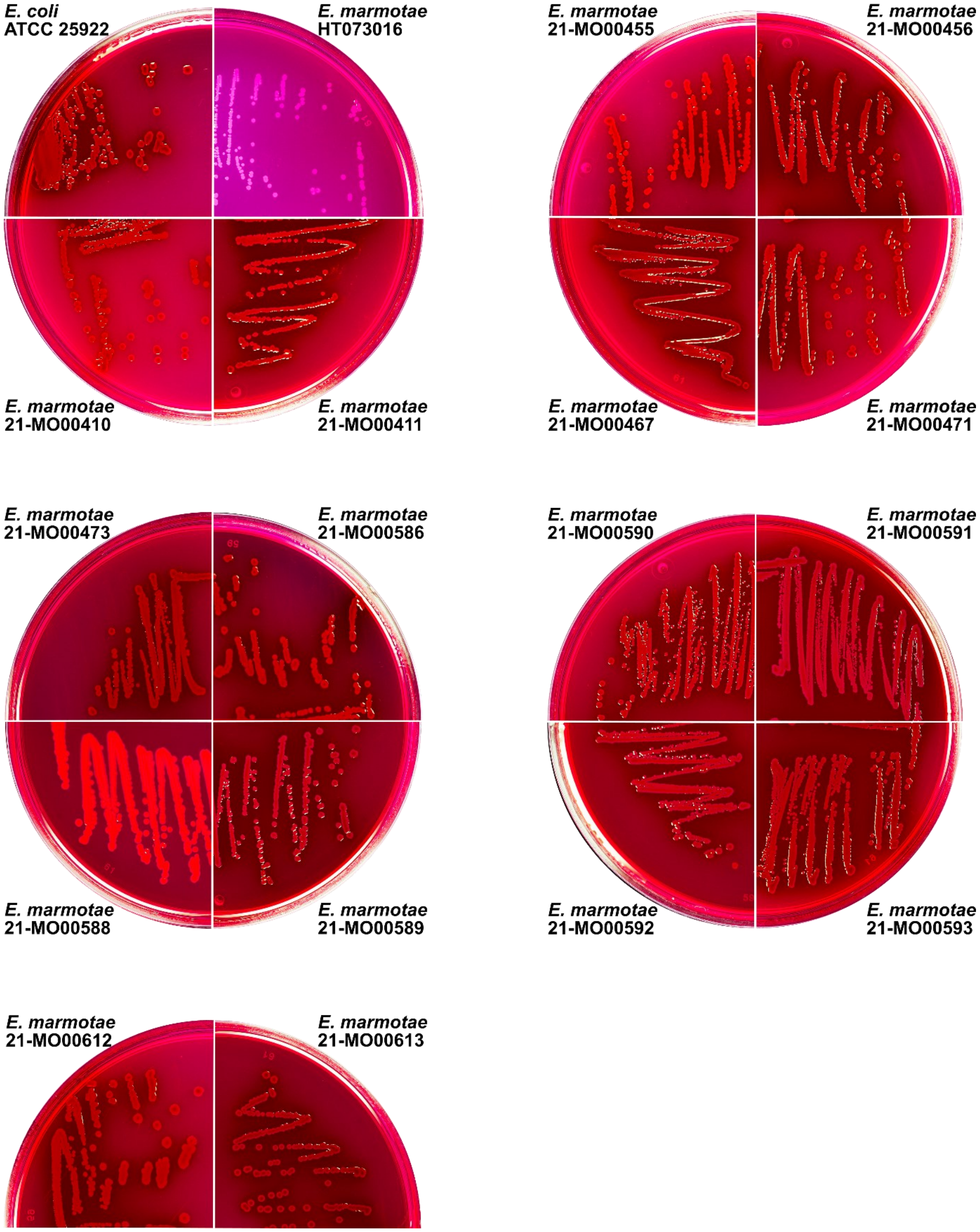
E. coli
ATCC 25922
E. marmotae
HT073016
E. marmotae
21-MO00410
E. marmotae
21-MO00411
E. marmotae
21-MO00455
E. marmotae
21-MO00456
E. marmotae
21-MO00467
E. marmotae
21-MO00471
E. marmotae
21-MO00473
E. marmotae
21-MO00586
E. marmotae
21-MO00588
E. marmotae
21-MO00589
E. marmotae
21-MO00590
E. marmotae
21-MO00591
E. marmotae
21-MO00592
E. marmotae
21-MO00593
E. marmotae
21-MO00612
E. marmotae
21-MO00613

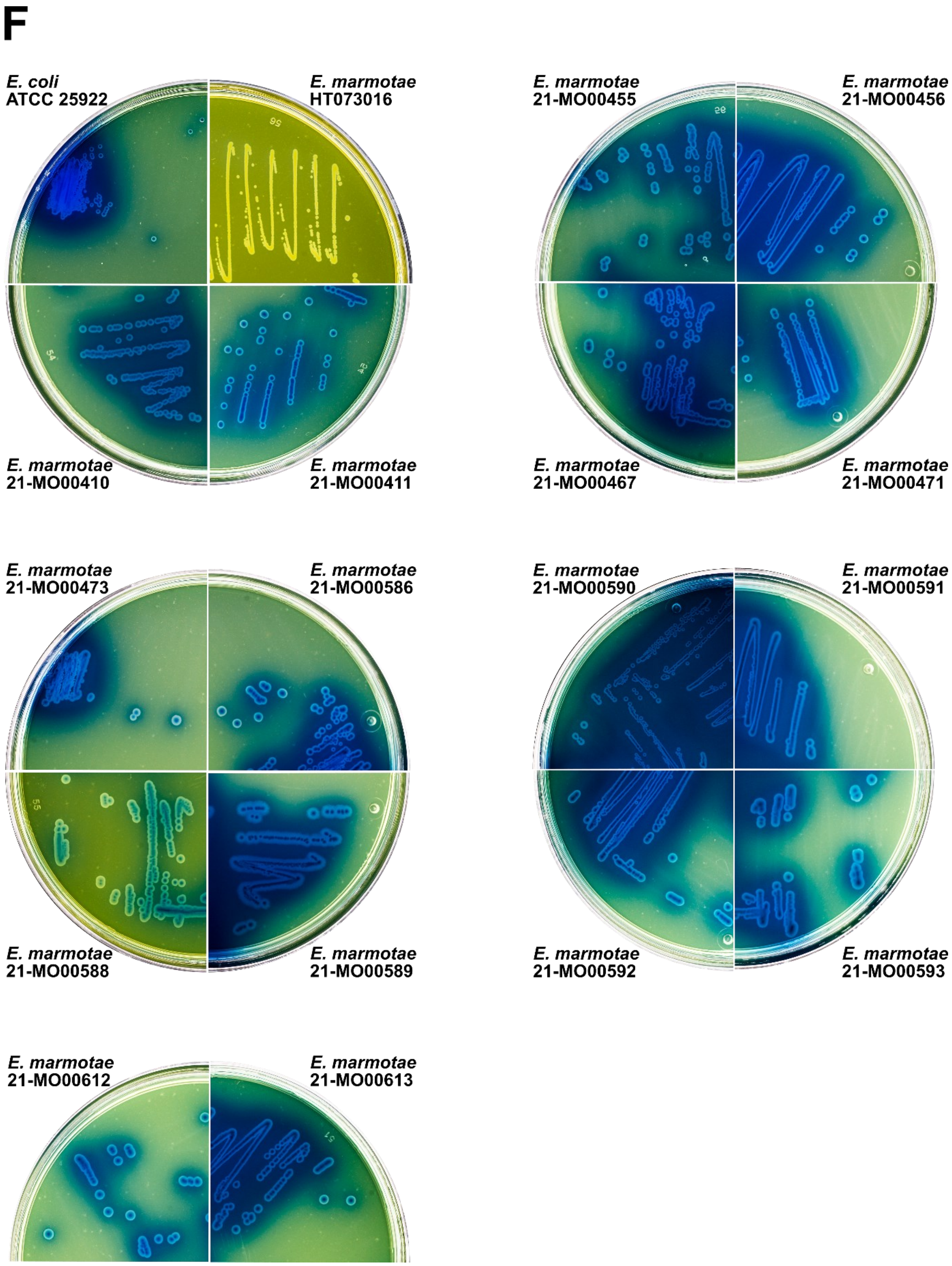

Figure S3: Assessment of phenotypic growth properties of *E. marmotae* on different culture media. *E. marmotae* isolates were grown at 37°C for 24 h on solid LB-agar plates (A), TSA-agar plates (B), TBX-agar plates (C), Columbia blood agar plates (D), Endo-agar plates (E), and Gassner agar plates (F). Figure S2 corresponds with Table S1.

Figure S4

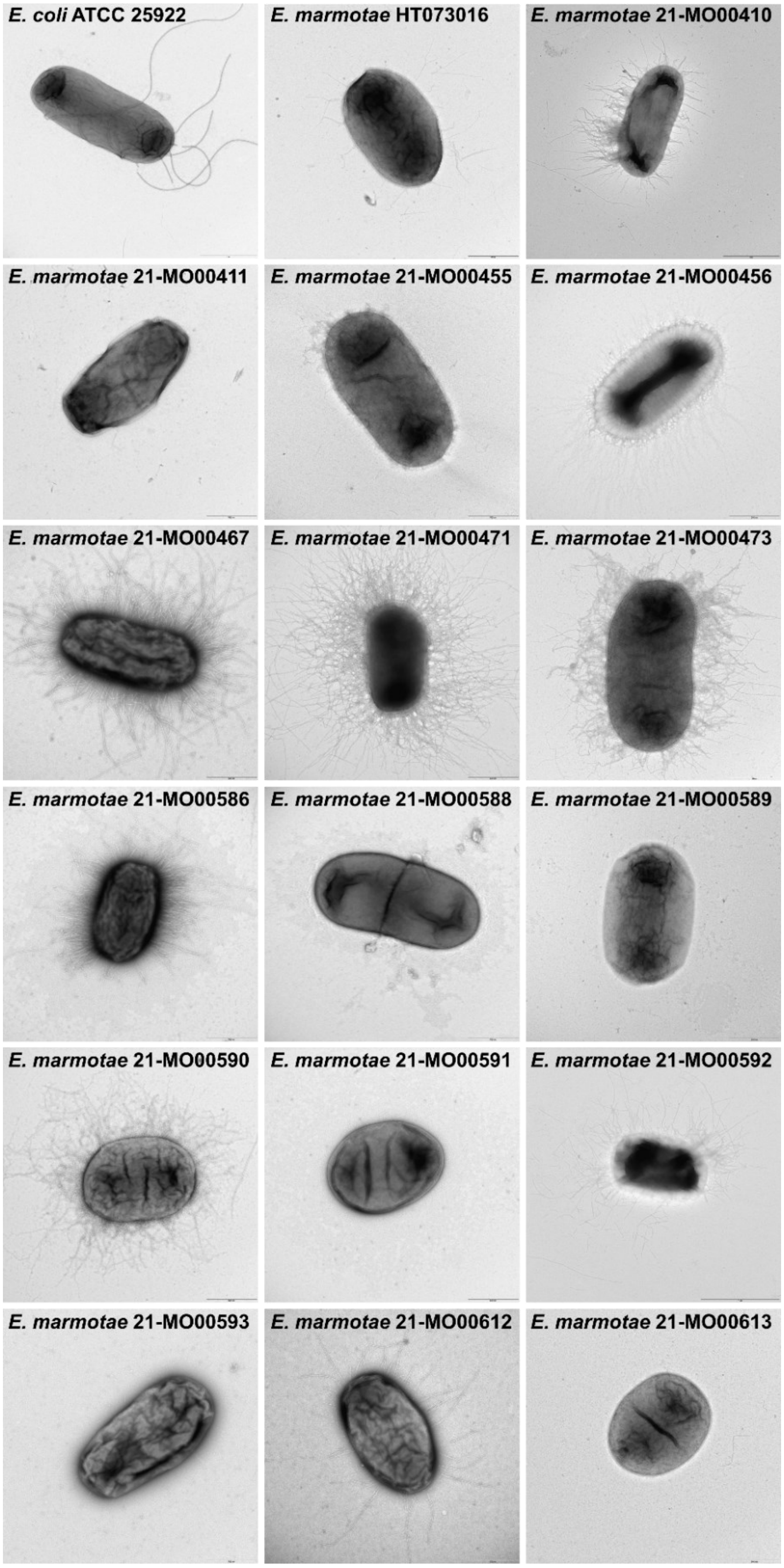

Figure S4: Transmission electron microscopy of German *E. marmotae* isolates. Phenotypic comparison of German *E. marmotae* isolates using *E. coli* ATCC 25922 and *E. marmotae* HT073016 as reference. Bacterial suspensions were added to carbon/formvar coated 400 mesh copper grids followed by fixation with 2.5% glutaraldehyde staining with 1% uranyl acetate. Imaging was performed on Jeol 1400 Plus transmission electron microscope (Jeol GmbH, Germany) operated at 120 kV.

Figure S5

	21-MO01158-0	21-MO01157-0	21-MO00411-0	21-MO00586-0	21-MO00612-1	21-MO00467-0	21-MO00456-0	21-MO00613-0	21-MO00471-0	21-MO01153-0	21-MO00625-0	21-MO00473-0	21-MO00410-0	21-MO01154-0	21-MO01161-0	21-MO00589-0	21-MO00588-0	21-MO00593-0	21-MO00455-0	21-MO00590-0	21-MO00592-0	21-MO01155-0	21-MO01160-0	21-MO01156-0	21-MO00591-0
**21-MO01158-0**	0	0	222	219	236	228	225	1306	2228	8826	9036	9133	9092	8805	9480	9319	8857	8894	9137	9138	9142	8926	8823	8734	8737
**21-MO01157-0**	0	0	222	219	236	228	225	1306	2228	8826	9036	9133	9092	8805	9480	9319	8857	8894	9137	9138	9142	8926	8823	8734	8737
**21-MO00411-0**	222	222	0	69	90	82	79	1300	2219	8803	9008	9107	9066	8771	9454	9296	8830	8867	9111	9108	9116	8898	8799	8707	8710
**21-MO00586-0**	219	219	69	0	79	71	68	1297	2216	8806	9011	9108	9067	8778	9455	9297	8837	8874	9114	9111	9119	8903	8800	8706	8709
**21-MO00612-1**	236	236	90	79	0	88	85	1314	2233	8823	9028	9125	9084	8795	9472	9314	8854	8891	9131	9128	9136	8922	8817	8725	8728
**21-MO00467-0**	228	228	82	71	88	0	69	1306	2225	8815	9020	9117	9076	8785	9464	9306	8846	8883	9120	9117	9125	8912	8808	8717	8720
**21-MO00456-0**	225	225	79	68	85	69	0	1303	2222	8812	9017	9114	9073	8784	9461	9301	8843	8880	9118	9115	9123	8911	8806	8714	8717
**21-MO00613-0**	1306	1306	1300	1297	1314	1306	1303	0	1529	8857	9098	9193	9152	8888	9559	9421	8946	8996	9214	9215	9219	8995	8924	8833	8834
**21-MO00471-0**	2228	2228	2219	2216	2233	2225	2222	1529	0	9855	10076	10171	10130	9869	10541	10396	9914	9971	10194	10193	10199	9992	9871	9805	9806
**21-MO01153-0**	8826	8826	8803	8806	8823	8815	8812	8857	9855	0	9381	9467	9442	9177	9866	9567	9130	9190	9364	9361	9355	9127	9152	8941	8948
**21-MO00625-0**	9036	9036	9008	9011	9028	9020	9017	9098	10076	9381	0	520	525	9159	9863	9485	9170	9270	9258	9257	9259	9148	9182	9027	9030
**21-MO00473-0**	9133	9133	9107	9108	9125	9117	9114	9193	10171	9467	520	0	105	9250	9945	9568	9260	9350	9349	9342	9344	9228	9281	9111	9108
**21-MO00410-0**	9092	9092	9066	9067	9084	9076	9073	9152	10130	9442	525	105	0	9219	9922	9553	9257	9339	9336	9329	9331	9211	9270	9098	9093
**21-MO01154-0**	8805	8805	8771	8778	8795	8785	8784	8888	9869	9177	9159	9250	9219	0	9600	9491	9034	9108	9200	9193	9191	9049	9067	8810	8817
**21-MO01161-0**	9480	9480	9454	9455	9472	9464	9461	9559	10541	9866	9863	9945	9922	9600	0	9280	9683	9690	9726	9713	9715	9777	9694	9666	9665
**21-MO00589-0**	9319	9319	9296	9297	9314	9306	9301	9421	10396	9567	9485	9568	9553	9491	9280	0	9419	9446	9482	9465	9469	9506	9321	9299	9296
**21-MO00588-0**	8857	8857	8830	8837	8854	8846	8843	8946	9914	9130	9170	9260	9257	9034	9683	9419	0	3689	9249	9238	9240	8901	8995	8851	8852
**21-MO00593-0**	8894	8894	8867	8874	8891	8883	8880	8996	9971	9190	9270	9350	9339	9108	9690	9446	3689	0	7842	7831	7837	9013	9114	8954	8961
**21-MO00455-0**	9137	9137	9111	9114	9131	9120	9118	9214	10194	9364	9258	9349	9336	9200	9726	9482	9249	7842	0	167	173	9261	9253	9106	9113
**21-MO00590-0**	9138	9138	9108	9111	9128	9117	9115	9215	10193	9361	9257	9342	9329	9193	9713	9465	9238	7831	167	0	146	9258	9248	9105	9108
**21-MO00592-0**	9142	9142	9116	9119	9136	9125	9123	9219	10199	9355	9259	9344	9331	9191	9715	9469	9240	7837	173	146	0	9260	9252	9103	9106
**21-MO01155-0**	8926	8926	8898	8903	8922	8912	8911	8995	9992	9127	9148	9228	9211	9049	9777	9506	8901	9013	9261	9258	9260	0	9033	8730	8735
**21-MO01160-0**	8823	8823	8799	8800	8817	8808	8806	8924	9871	9152	9182	9281	9270	9067	9694	9321	8995	9114	9253	9248	9252	9033	0	8741	8749
**21-MO01156-0**	8734	8734	8707	8706	8725	8717	8714	8833	9805	8941	9027	9111	9098	8810	9666	9299	8851	8954	9106	9105	9103	8730	8741	0	77
**21-MO00591-0**	8737	8737	8710	8709	8728	8720	8717	8834	9806	8948	9030	9108	9093	8817	9665	9296	8852	8961	9113	9108	9106	8735	8749	77	0

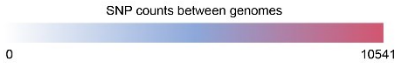

Figure S5: SNP matrix obtained by phylogenetic comparisons of German *E. marmotae* isolates. SNPs was calculated using CSIPhylogeny 1.4 under default settings and the exclusion of heterozygous SNPs. A) Single nucleotide polymorphisms (SNPs) were called by mapping the genomes of the German isolates to the genome of 21-MO00411 as reference.

Supplemental Material

Figure S6

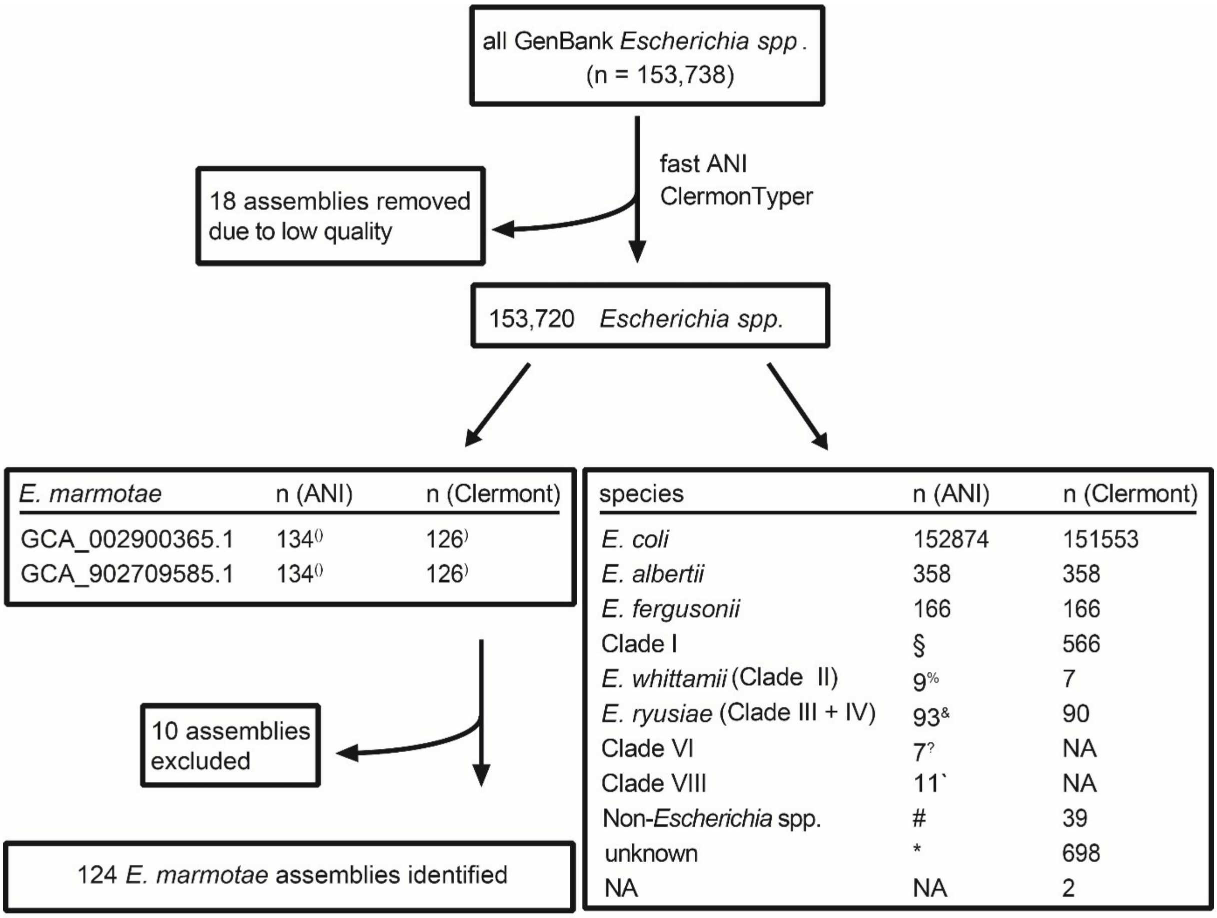

§ all assemblies matched also with *E. coli* reference with ANI >95%

% 2 assemblies matched also with *E. coli* reference with ANI >95%

& 3 assemblies assigned as *E. coli* by ClermonTyper

? 3 assemblies matched also with *E. coli* reference with ANI >95%;
6 assemblies assigned as *E. coli* by ClermonTyper

` 2 assemblies matched also with *E. coli* reference with ANI >95%;
10 assemblies assigned as *E. coli* by ClermonTyper

# match with no reference/several references

* match with several references

(8 assemblies matched with several references with ANI >95%

) one isolate had 2 different accesion numbers, and one iolate was excluded due to low quality

NA not available

Figure S6: Flow Chart of *E. marmotae* identification strategy.

Figure S7

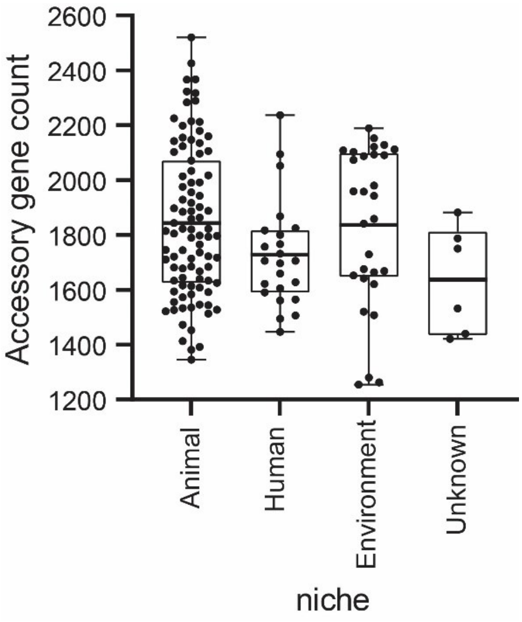

Figure S7: Number of accessory genes in *E. marmotae* isolates from different sources. Number of accessory genes are shown as scatter dot plot and box plot including median and whiskers from minimum to maximum. Differences between medians of different niches are not significant using a Kruskal-Wallis test with Dunn's multiple comparisons test.

Supplemental Material

Figure S8

a

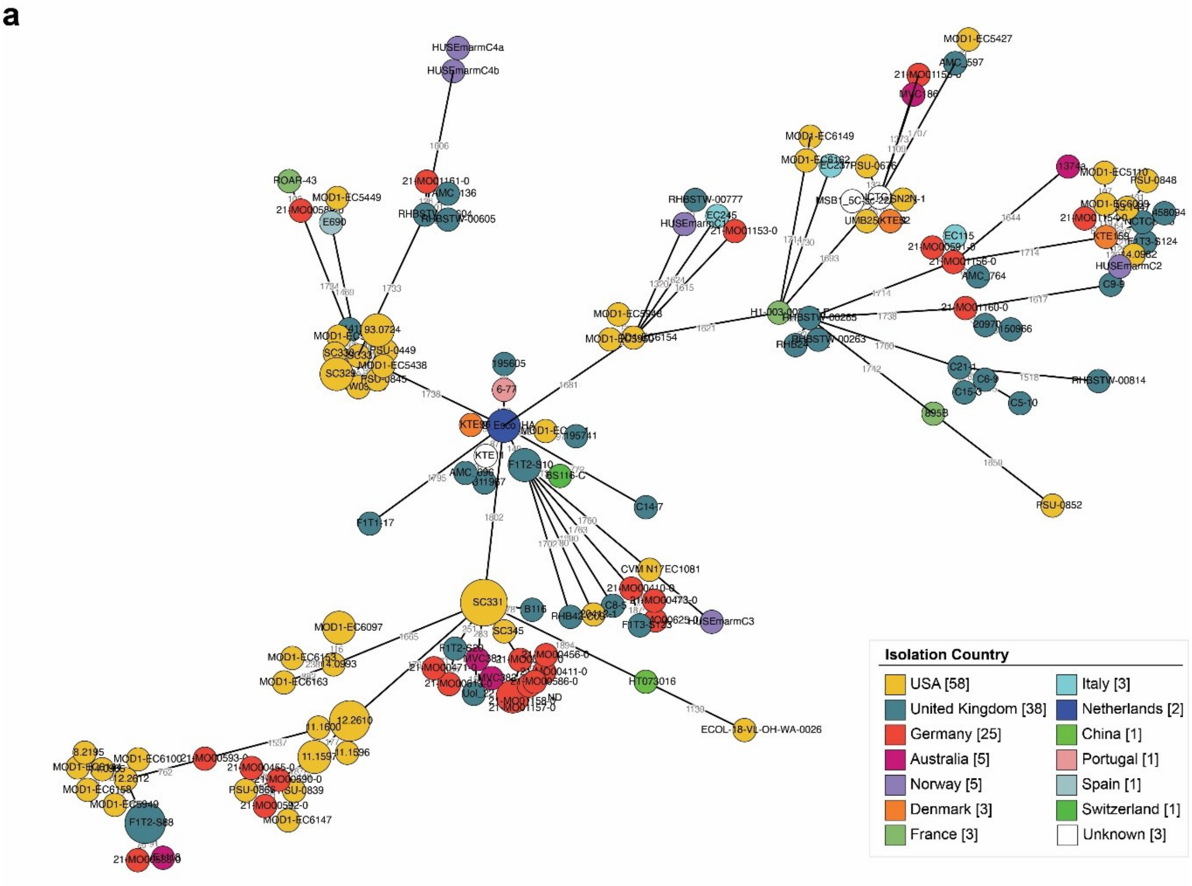

b

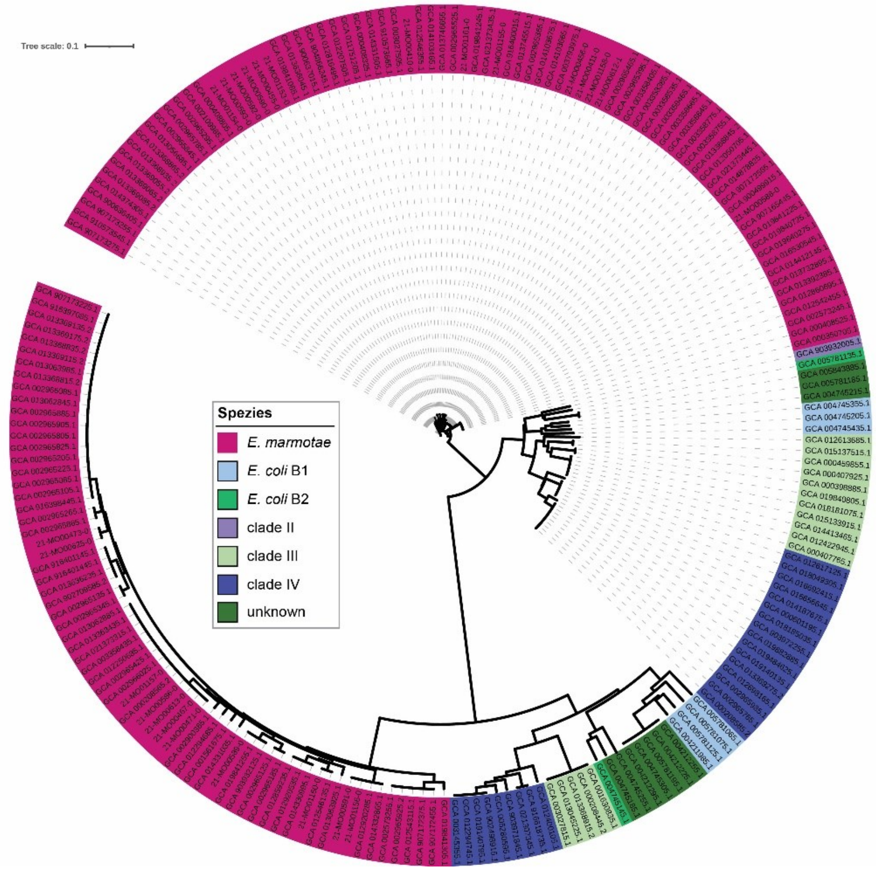

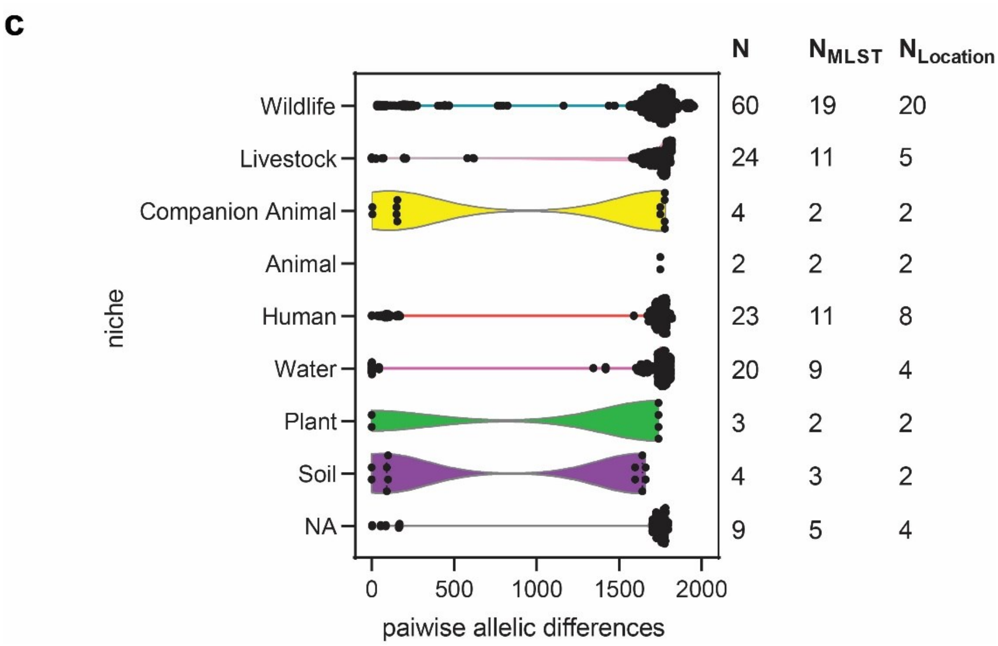

Figure S8: Genomic diversity of *E. marmotae* with regard to isolation country, H-antigen and niche. a, Minimum spanning tree of 149 *E. marmotae* isolates using the *E. coli* scheme from EnteroBase. Each node represents a distinct cgMLST. The size of each node indicates the number of isolates within that node. Isolates are colored according to their isolation country. **b**, Phylogram of the nucleotide sequence of the antigen H56 of 149 *E. marmotae* and 61 non-*E. marmotae* isolates. **c**, Comparison of pairwise allelic differences (PADs) within different niches based on individual cgMLSTs. Violin plots show probability densities of PADs. N: number of isolates within that niche. N_{MLST}: Number of different MLST within the niche. $N_{Location}$: Number of different sites where *E. marmotae* was isolated.

Figure S9

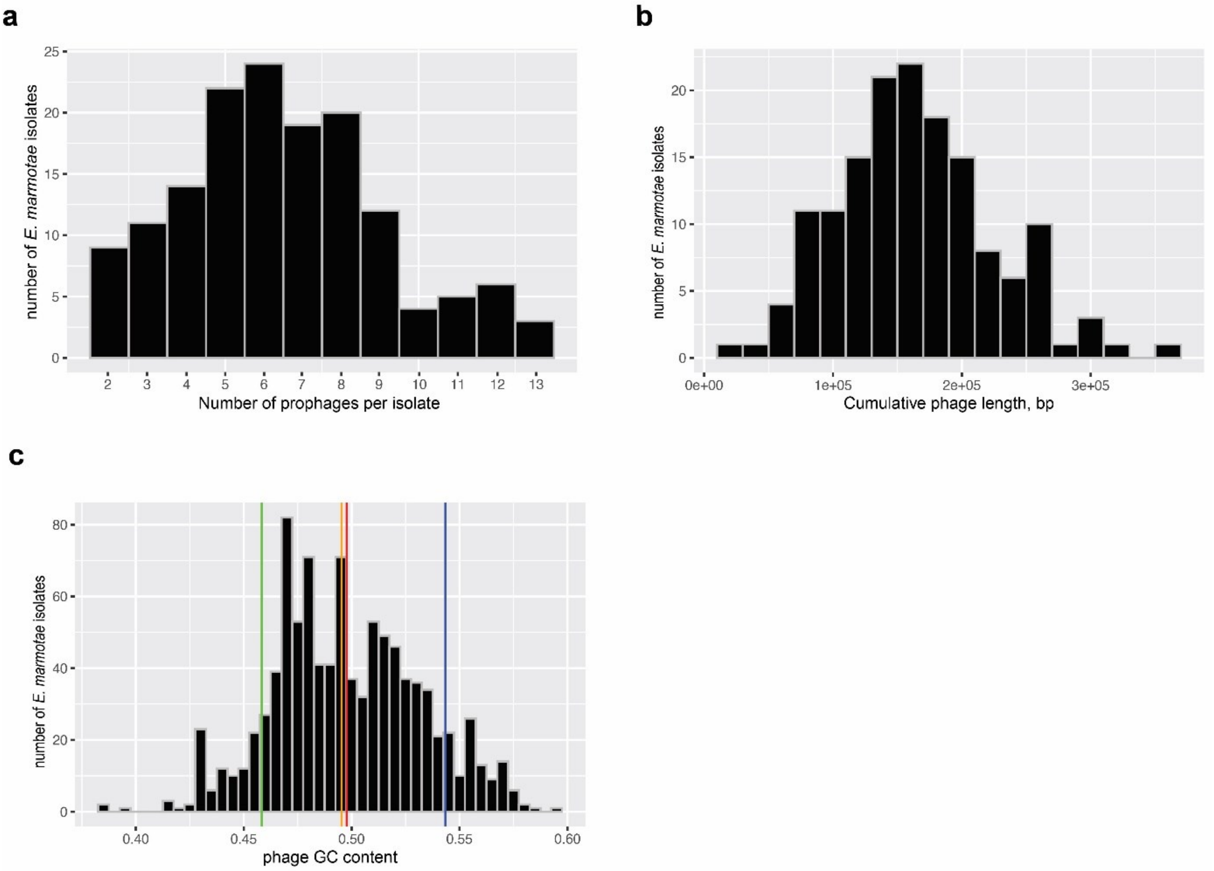

Figure S9: Analysis of prophages in the *E. marmotae* population. a, Distribution of the total number of prophages per *E. marmotae* isolate. **b**, Cumulative length [bp] of prophages per *E. marmotae* isolate. **c**, Histogram of the GC content of prophages. The colored lines in represent the mean GC content of the phage sequences (red), the median (orange), and the 0.1 and 0.9 quantiles (green and blue, respectively).

Figure S10

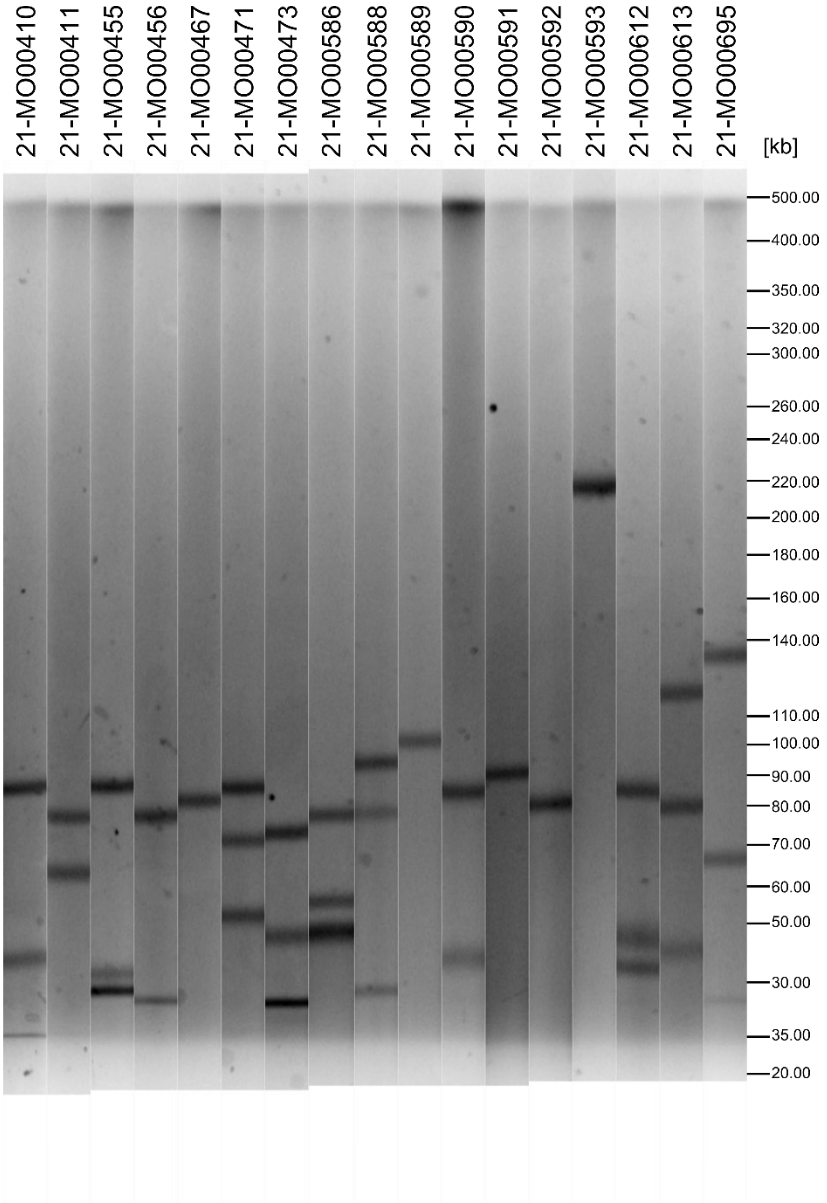

Figure S10: S1-pulsed-field gel electrophoresis of German *E. marmotae* isolates. Plasmid profiles of the German *E. marmotae* isolates were obtained by PFGE using the S1 restriction endonuclease as previously described (8). PFGE analysis was conducted with Bionumerics 7.6.3 (Applied Maths, Sint-Martens-Latem; Belgium).

Supplemental Table Legends

Table S1: Extended metadata of 149 *E. marmotae* isolates.

Table S2: Phenotypic and biochemical characteristics of *E. marmotae* isolates.

Table S3: Antimicrobial susceptibility testing of German *E. marmotae* isolates.

Table S4: Virulence factors present in *E. marmotae*.

Table S5: Prediction of mobile genetic elements.

Table S6: Reference plasmid prediction for AMR-gene carrying plasmids of 12 *E. marmotae* isolates.

Table S7: Bakta annotation of 149 *E. marmotae* isolates.

References

1. Binsker U, Oelgeschläger K, Neumann B, Werner G, Käsbohrer A, Hammerl JA. Genomic Evidence of mcr-1.26 IncX4 Plasmid Transmission between Poultry and Humans. Microbiology Spectrum. 2023;11(4).
2. Deneke C, Brendebach H, Uelze L, Borowiak M, Malorny B, Tausch SH. Species-Specific Quality Control, Assembly and Contamination Detection in Microbial Isolate Sequences with AQUAMIS. Genes-Basel. 2021;12(5).
3. Schwartz K, Borowiak M, Deneke C, Balau V, Metelmann C, Strauch E. Complete and Circularized Genome Assembly of a Human Isolate of Vibrio navarrensis Biotype pommerensis with MiSeq and MinION Sequence Data. Microbiol Resour Ann. 2021;10(5).
4. Beghain J, Bridier-Nahmias A, Le Nagard H, Denamur E, Clermont O. ClermonTyping: an easy-to-use and accurate in silico method for Escherichia genus strain phylotyping. Microb Genomics. 2018;4(7).
5. Kaas RS, Leekitcharoenphon P, Aarestrup FM, Lund O. Solving the Problem of Comparing Whole Bacterial Genomes across Different Sequencing Platforms. Plos One. 2014;9(8).
6. Munck N, Leekitcharoenphon P, Litrup E, Kaas R, Meinen A, Guillier L, et al. Four European Salmonella Typhimurium datasets collected to develop WGS-based source attribution methods. Sci Data. 2020;7(1):75.
7. Clermont O, Christenson JK, Denamur E, Gordon DM. The Clermont Escherichia coli phylo-typing method revisited: improvement of specificity and detection of new phylo-groups. Env Microbiol Rep. 2013;5(1):58-65.
8. Alfredo Caprioli AM, Valeria Michelacci, Stefano Morabito. Molecular typing of Verocytotoxin-producing E. coli (VTEC) strains isolated from food, feed and animals: state of play and standard operating procedures for pulsed field gel electrophoresis (PFGE) typing, profiles interpretation and curation. EFSA supporting publication. 2014;2014:EN-704, 55 pp. .
9. Falkenhagen A, Patzina-Mehling C, Gadicherla AK, Strydom A, O'Neill HG, Johne R. Generation of Simian Rotavirus Reassortants with VP4-and VP7-Encoding Genome Segments from Human Strains Circulating in Africa Using Reverse Genetics. Viruses-Basel. 2020;12(2).
10. Juraschek K, Deneke C, Schmoger S, Grobbel M, Malorny B, Kasbohrer A, et al. Phenotypic and Genotypic Properties of Fluoroquinolone-Resistant, qnr-Carrying Escherichia coli Isolated from the German Food Chain in 2017. Microorganisms. 2021;9(6).
